# Expression of *Streptococcus pneumoniae* Bacteriocins Is Induced by Antibiotics via Regulatory Interplay with the Competence System

**DOI:** 10.1371/journal.ppat.1005422

**Published:** 2016-02-03

**Authors:** Morten Kjos, Eric Miller, Jelle Slager, Frank B. Lake, Oliver Gericke, Ian S. Roberts, Daniel E. Rozen, Jan-Willem Veening

**Affiliations:** 1 Molecular Genetics Group, Groningen Biomolecular Sciences and Biotechnology Institute, Centre for Synthetic Biology, University of Groningen, Groningen, The Netherlands; 2 Institute of Biology, Leiden University, Leiden, the Netherlands; 3 Faculty of Life Sciences, University of Manchester, Manchester, United Kingdom; University of Tubingen, GERMANY

## Abstract

Pneumococcal bacteriocins (pneumocins) are antibacterial toxins that mediate intra-species competition within the human host. However, the triggers of pneumocin expression are poorly understood. Using RNA-sequencing, we mapped the regulon of the pneumocin cluster (*blp*) of *Streptococcus pneumoniae* D39. Furthermore, by analogy with pneumococcal competence, we show that several antibiotics activate the *blp*-genes. Using real-time gene expression measurements we show that while the promoter driving expression of the two-component regulatory system *blpR/H* is constitutive, the remaining *blp*-promoters that control pneumocin expression, immunity and the inducer peptide BlpC, are pH-dependent and induced in the late exponential phase. Intriguingly, competence for genetic transformation, mediated by the paralogous ComD/E two-component quorum system, is induced by the same environmental cues. To test for interplay between these regulatory systems, we quantified the regulatory response to the addition of synthetic BlpC and competence-stimulating peptide (CSP). Supporting the idea of such interplay, we found that immediately upon addition of CSP, the *blp*-promoters were activated in a *comD/E*-dependent manner. After a delay, *blp*-expression was highly induced and was strictly dependent on *blpRH* and *blpC*. This raised the question of the mechanism of BlpC export, since bioinformatic analysis showed that the genes encoding the putative exporter for BlpC, *blpAB*, are not intact in strain D39 and most other strains. By contrast, all sequenced pneumococcal strains contain intact *comAB* genes, encoding the transport system for CSP. Consistent with the idea that *comAB* mediate BlpC export, we finally show that high-level expression of the *blp*-genes requires *comAB*. Together, our results demonstrate that regulation of pneumocin expression is intertwined with competence, explaining why certain antibiotics induce *blp*-expression. Antibiotic-induced pneumocin expression might therefore have unpredictable consequences on pneumococcal colonization dynamics by activating genes that mediate intra-specific interference competition.

## Author Summary

*Streptococcus pneumoniae* is an opportunistic pathogen with high carriage rates in children. Pneumococci express pneumocins that kill competing bacteria. Pneumocin expression is controlled by a pheromone-induced two-component system (BlpR/H) but the triggers for the system are poorly understood. We show that the pheromone-induced two-component system driving competence for genetic transformation, ComD/E, also controls expression of BlpC, the peptide pheromone activating BlpR/H-dependent gene expression. Importantly, we show that the competence pheromone exporter, ComAB, also exports BlpC. Since antibiotics that disrupt protein quality control or DNA replication trigger competence, it follows that the same antibiotics activate pneumocin expression. Our experiments show that this dual-quorum sensing system ensures that pneumocins are expressed at the end of exponential growth when nutrients become limiting. Pneumocin expression might thus be used to liberate nutrients by lysing competing bacteria. Antibiotic-induced pneumocin production might also aid in clearing the niche after antibiotic stress. Any free DNA can then be used for transformation to acquire antibiotic-resistance.

## Introduction

*Streptococcus pneumoniae* is a Gram-positive opportunistic pathogen that resides in the human nasopharynx. Pneumococci can cause invasive and non-invasive infections to which children, the elderly and the immunocompromised are particularly susceptible. The carriage rate of *S*. *pneumoniae* in the human population can be very high. Up to 80% of children under the age of 5 are colonized [1], and colonization with multiple strains simultaneously is widespread. Competition between strains in the human nasopharynx during co-colonization has important implications for the epidemiology of the pneumococcus, potentially influencing strain prevalence, serotype distributions and disease progression.

Among the most important potential drivers of intraspecific competition are several diverse classes of pneumococcal bacteriocins (small antimicrobial peptides), including the CibAB two-peptide bacteriocin [2], a lantibiotic [3] and the Blp bacteriocins (pneumocins) [4–6]. The *blp* locus is ubiquitous in pneumococcal genomes, and the operon is exceptionally diverse, suggesting that these toxins have evolved via diversifying selection. Puzzlingly, however, in most pneumococcal strains the genes mediating export of the *blp* peptides, encoding the ABC transporter BlpAB, carry frameshift mutation(s) that render these genes non-functional. This paradox raises two questions: 1) are Blp bacteriocins exported in strains lacking a functional transporter? and 2) if so, by which mechanism does this occur? One possibility is that bacteriocins are not exported, but that strains with interrupted *blpAB* alleles constitute so-called cheater sub-populations, which cannot export pneumocins alone but are able to express immunity in response to co-colonizing strains [7]. Such cheaters would therefore only activate *blp* expression in response to secreted BlpC in their surroundings. A second possibility is that Blp bacteriocins have an alternative mode of export and that strains with non-functional BlpAB transporters can activate their own *blp* expression. Here we provide direct evidence for this second hypothesis and show that *blp* bacteriocins are co-regulated with competence for transformation. Moreover, we show that environmental cues regulating competence overlap with those that induce Blp secretion. These functional data clarify the mechanisms of *blp* regulation and cast doubt on the validity of the *blpA* cheater hypothesis.

Genes for the production and regulation of Blp bacteriocins are organized in gene clusters with several operons, typically flanked by the genes *ecsB* and a putative choline kinase located at approximately 400–600 kb from the origin of replication. Pneumocin expression is regulated by a classical quorum sensing two-component regulatory system [5,8] which is conserved among diverse *S*. *pneumoniae* genomes [9]. The inducer peptide BlpC, which contains an N-terminal double-glycine leader sequence, is putatively processed and exported by an ABC transporter system, BlpAB. Indeed, Kochan and Dawid showed that an engineered laboratory strain with intact BlpAB efficiently processes BlpC but not in the absence of BlpA [10]. When the external concentration of BlpC exceeds a certain threshold, it binds specifically to a membrane-located histidine protein kinase BlpH [9], which activates the DNA binding response regulator BlpR by phosphorylation (BlpR-P). BlpR-P binds to specific sequence sites in the promoter region of *blp* to activate their expression. In addition to genes for regulation and transport, the *blp* gene cluster of *S*. *pneumoniae* also contains two genes of unknown function (*blpS* and *blpT*) along with genes encoding (putative) pneumocins (known as *blpD*, *blpE*, *blpI*, *blpJ*, *blpK*, *blpM*, *blpN*, *blpO*, *blpQ*, *pncT* and *pncW*) and cognate immunity genes (known as *blpL*, *blpX*, *blpY*, *blpZ* and *pncP*) [11]. Similar to BlpC, the pneumocins contain an N-terminal double-glycine leader, which is processed upon export via the ABC-transporter system. The region encoding pneumocins and immunity proteins (referred to as the BIR, bacteriocin immunity region) is highly variable and the number of pneumocin genes differs greatly between strains [7,12]. However, despite significant variation in the BIR region, a superficial analysis indicates that the cluster is intact even in strains with a degenerated *blpA*.

A likely candidate mechanism for Blp peptide export is the paralogous quorum-based two-component signaling system regulating competence for natural transformation (*comCDE*). *com* regulation is mediated by the export of a quorum sensing peptide (CSP) via an ABC transporter ComAB, followed by CSP concentration-dependent activation of downstream late competence genes. Importantly, the *blp* genes have been shown to be weakly upregulated during pneumococcus competence [13]. In addition to the Blp bacteriocins, competent *S*. *pneumoniae* also express CbpD and CibAB, a murein hydrolase and a two-peptide bacteriocin, respectively, which both cause lysis of non-competent cells [2].

The competence regulatory system is highly sensitive to environmental cues such as pH and exposure to certain antibiotics [14–16]. For example, sub-MIC concentrations of antibiotics that perturb DNA replication elongation (e.g. ciprofloxacin, trimethoprim and mitomycin C), induce competence by increasing the gene dosage of the *comCDE* genes, which are located close to the replication origin [16]. Moreover, aminoglycosides (e.g. kanamycin and streptomycin), which target protein synthesis and lead to high numbers of misfolded proteins, induce competence by decreasing the degradation rate of CSP in the external environment. This occurs because in cells exposed to aminoglycosides, the protease HtrA, which normally degrades CSP, is occupied with targeting misfolded proteins [17].

Our aims here are twofold. First, we critically evaluate hypotheses derived from the proposal that strains carrying degenerate *blpA* genes are cheaters that lack Blp secretion. Secondly, failing to find support for this idea, we seek mechanisms that could offset the consequences of *blpA* lesions, focusing in particular on the roles of the paralogous *com* operon and the influence of environmental cues. Briefly, we demonstrate that *blp* expression is co-regulated with the competence regulatory system of *S*. *pneumoniae* and show that the ‘cheaters’-hypothesis [7] is inconsistent with both bioinformatics and experimental data.

## Results

### Strains with degenerate *blpAB* do not lose *blp* bacteriocins

**Fig 1:**
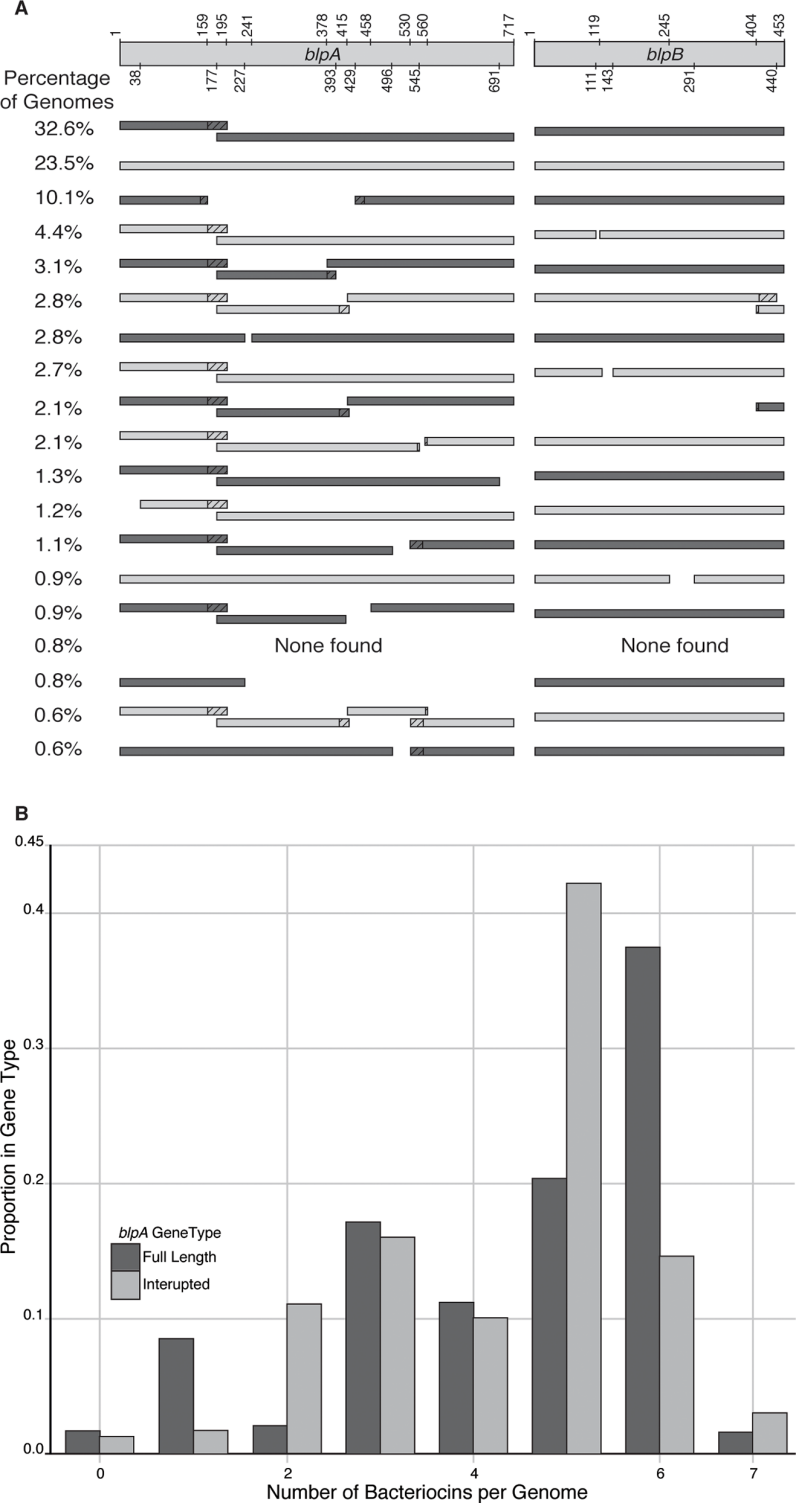
The *blpAB* genes are frameshifted in the majority of pneumococcal genomes. (A) Distribution of *blpAB* ORF lengths in a set of 4,096 genomes. Amino acid residue numbers are shown at the top. Variants found in less than 0.5% of the genomes are not shown. Frameshifted regions encoding non-BlpA ORFs are shown as diagonal lines. (B) Distribution of bacteriocins between genomes. The proportion of genomes with different numbers of *blp* bacteriocins for genomes with a full-length *blpA* (n = 1,020) is compared to genomes with an interrupted *blpA* (n = 3,028). No *blpA* sequence was detected in 48 genomes.

We identified *blp* bacteriocin open reading frames (ORFs) in 4,096 *S*. *pneumoniae* genomes, as well as all ORFs that had reciprocal best BLAST hits to *blpA*, *blpB*, *comA* and *comB*. We identified striking diversity in the length of *blpA/B* across these genomes, with only 23.5% of genomes containing full-length *blpA* and *blpB* sequences (Fig 1A). We found a large diversity of indel and stop codon mutations that lead to interrupted ORFs in both of these genes. The entire *blpAB* region is completely deleted along with the adjoining *blpC* in 21 strains, while an additional 27 strains have no *blpAB* but still contain *blpC*. Thus, the majority of pneumococcal strains retain *blpAB* pseudogenes in the genome. If strains lacking a functional *blpAB* are incapable of exporting bacteriocins, then we would predict that these strains would lose bacteriocin genes by genetic drift. However, we only found a difference of 0.14 bacteriocins between the genomes with a full-length *blpA* (average = 4.46 bacteriocins) compared to the number of bacteriocins found in genomes with interrupted *blpA* ORFs (average = 4.32 bacteriocins; Wilcoxon rank test, 1.55 x 10^−8^; Fig 1B). Although non-functional bacteriocin genes may be retained in the genome, this small difference in average number of bacteriocins between genomes with and without *blpA* suggests that bacteriocin secretion is not exclusively dependent on a functional BlpAB.

**Fig 2:**
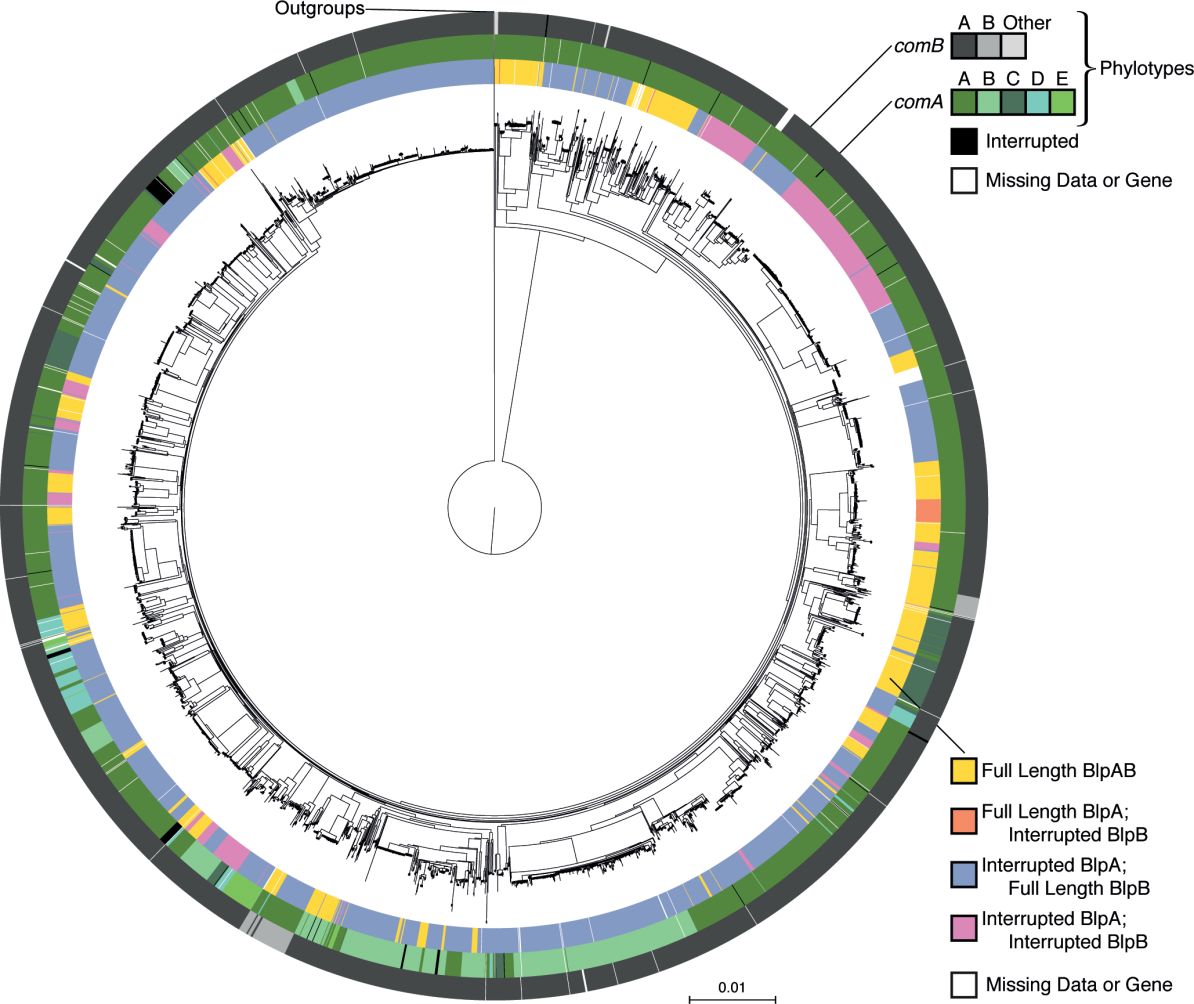
Association between *blpAB* length and *comAB* phylotypes. The phylogenetic tree of *S*. *pneumoniae* genomes is surrounded by three rings; moving outwards, these show the ORF lengths for *blpAB*, and the phylotypes for *comA* and *comB*, respectively. Partial length ORFs and missing data are also shown for *comA* and *comB*. *comB* phylotypes found in less than 0.5% of genomes are colored grey to indicate their status as full-length variants.

In order to examine the co-occurrence of interrupted ORFs in *blpA* and *blpB*, we mapped these gene variants onto a phylogenetic tree assembled using full genome SNPs (Fig 2). Using this phylogeny and a maximum likelihood approach, we found that genomes with an interrupted *blpA* are more likely to have an interrupted *blpB*, even after accounting for phylogeny (log ratio test, p = 7.86 x 10^−4^).

The ABC transporter system involved in competence, *comAB*, is the closest homolog to *blpAB* in *S*. *pneumoniae*. To examine if *comA* or *comB* variation is linked to co-occurrence of either full-length or interrupted ORFs of *blpA* and *blpB*, we divided *comA* and *comB* amino acid variants into similarity-based clusters termed phylotypes. We found no association between the phylotypes of either *comA* or *comB* and the structure of either *blpA* (log ratio test, p > 0.133 for *comA*, p > 0.139 for *comB*) or *blpB* (log ratio test, p > 0.0889 for *comA*, p > 0.537 for *comB*).

### Expression of *blp* genes in *S*. *pneumoniae* D39 is induced by antibiotics and is pH-dependent

The bioinformatics results strongly suggest that pneumococcal strains with degenerate *blpAB* alleles are still able to express pneumocins but it remains unknown what factors stimulate natural *blp* expression. Exposure to antibiotics can lead to global changes in gene expression and can induce the competent state in *S*. *pneumoniae* [14–16]. To test whether antibiotics also trigger pneumocin gene expression, we re-examined our earlier RNA-seq data [16] of *S*. *pneumoniae* D39 cells treated with sub-MIC levels of HPUra (6-(p-hydroxyphenylazo)-uracil) which blocks DNA replication [18] and kanamycin, which induces mistranslation [17]. In the absence of these agents, the *blp* genes were not expressed in C+Y medium at pH 7.4 (S1 Table). However, under the same pH conditions, both HPura and kanamycin weakly induced expression of some *blp* genes (S1 Table). To test if other antibiotics also induce *blp* gene expression, we performed transcriptome sequencing on RNA isolated from cells treated with ciprofloxacin (topoisomerase IV/DNA-gyrase inhibitor), hydroyxurea (decreases the cellular pool of dNTP via inhibition of ribonucleotide reductase) and rifampicin (RNA polymerase inhibitor). Similar to the earlier antibiotic exposure experiments [16], cells were harvested for RNA sequencing in early exponential phase. As previously observed, competence was activated by ciprofloxacin and hydroxyurea but not by rifampicin (S1 Table) [16]. Strikingly, ciprofloxacin and hydroxyurea also induced expression of some *blp* genes, although to a lesser degree (S1 Table). Together, the RNA-seq data shows that the same antibiotics that trigger competence also activate expression of *blp* genes.

**Fig 3:**
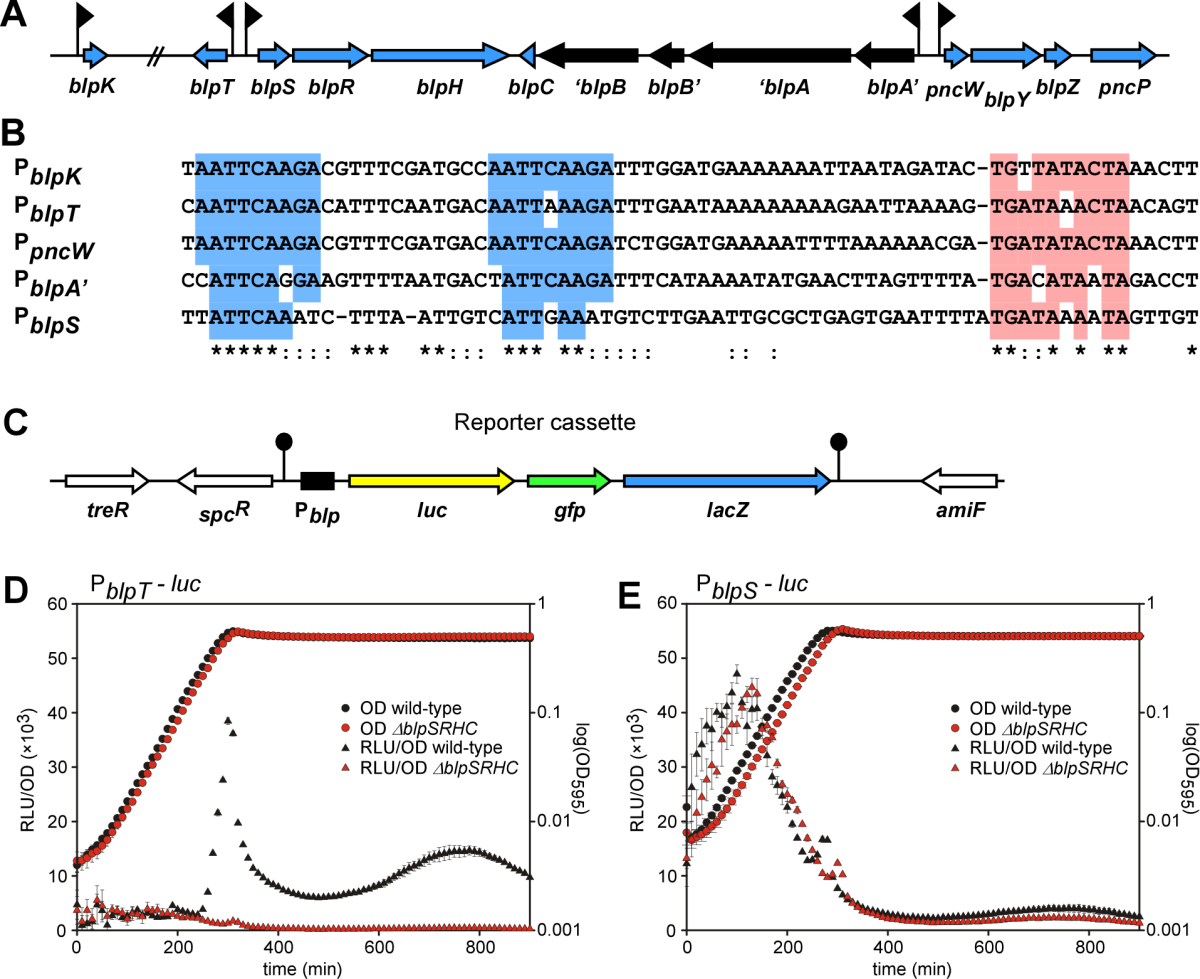
*blp* regulation in *S*. *pneumoniae* D39. (A) Schematic representation of the *blp* regulated genes of *S*. *pneumoniae* D39. The genes are represented by blue arrows and drawn to scale. Promoters are indicated by flags. *blpA* and *blpB* have frameshift mutations, as marked in black. Note that the ORF named *pncW* in this figure, encoding a putative bacteriocin upstream of *blpY*, is not annotated in the published genome sequence of *S*. *pneumoniae* D39. (B) Alignment of *blp* promoters. Nucleotides conserved in all five promoters are marked with an asterisk (*), while nucleotides conserved in 4 out of 5 promoters are marked with two dots (:). The direct repeats are shown in blue and the extended -10 box in red. (C) Schematic overview of the tripartite reporter cassette integrated in the *cep*-locus of *S*. *pneumoniae* D39 between *treR* and *amiF* [20]. The *blp* promoters (denoted P_*blp*_ in the text) are inserted upstream of the three co-transcribed reporters *luc* (yellow), *gfp* (green) and *lacZ* (blue). The reporter cassette is transcriptionally isolated by terminators (lollipops). (D) Natural induction of promoter P_*blpT*_. *S*. *pneumoniae* containing a fusion of promoter P_*blpT*_ to the reporter cassette *luc-gfp-lacZ*, grown in C+Y pH 8. Strains with or without deletion of the regulatory genes, *blpSRHC*, are shown. Natural expression from promoters P_*blpA*_, P_*pncW*_ and P_*blpK*_ are shown in S1 Fig. (E) P_*blpS*_ is active in all growth stages and expression is independent of the *blp* regulatory genes. *S*. *pneumoniae* D39 containing a fusion of promoter P_*blpS*_ to the reporter cassette *luc-gfp-lacZ*, grown in C+Y pH 8. For D and E, gene expression as measured by luciferase activity (relative luminescence units per optical density, RLU/OD) is shown on the left axis and growth as measured by absorbance at 595 nm (OD_595_) is shown on the right axis. Averages of 3 replicates with the standard deviation are plotted.

The *blp* gene cluster of strain D39 contains four transcriptional units [5]; the regulatory operon *blpSRH* (promoter P_*blpS*_), the transport operon *blpABC* (promoter P_*blpA*_), the bacteriocin/immunity operon *pncW-blpYZ-pncP* (promoter P_*pncW*_) and finally a transcriptional unit expressing a single gene of unknown function, *blpT* (promoter P_*blpT*_) (Fig 3A). In addition, another *blp* promoter (P_*blpK*_) is located outside the *blp* gene cluster in *S*. *pneumoniae* D39 (Fig 3A) [19] and controls the expression of BlpK, which encodes a putative bacteriocin. Each of the transcriptional units is likely under control by BlpR since the promoters contain an extended -10 element and upstream 9-bp tandem direct repeats (Fig 3B) [5]. Of the five promoters, P_*blpS*_ is the least conserved compared to P_*pncW*_, P_*blpT*_, P_*blpA*_ and P_*blpK*_.

In order to investigate the conditions that induce *blp* expression, we developed a novel tripartite reporter cassette containing firefly luciferase (*luc*), superfolder gfp (*gfp*) and β-galactosidase (*lacZ*) in the BglBrick-compatible vector pPEP1 [20] (Fig 3C). Using these different reporters, expression can be monitored in cultures through time (*luc*), at the single cell level (*gfp*) or in colonies on agar plates (*lacZ*). Each of the five *blp* promoters identified above were fused upstream of this cassette, and the reporter constructs were chromosomally integrated at an ectopic locus (Fig 3C and Materials and Methods).

Using *luc* we could follow expression of the *blp* promoters in a time-resolved manner. *S*. *pneumoniae* was grown in C+Y medium and expression was monitored by *luc* activity. Expression was observed from all five *blp* promoters at pH 8.0 but not at pH 7.0 (see below). For four of the promoters, P_*blpT*_, P_*blpA*_, P_*pncW*_ and P_*blpK*_, expression was activated simultaneously in late exponential phase and switched off when the population reached stationary phase (Fig 3D, S1 Fig). A second shallow peak of expression was observed in late stationary phase (Fig 3D, S1 Fig). Interestingly, P_*blpS*_, encoding the regulatory system, was active from early exponential phase, and displayed expression dynamics similar to known constitutive promoters in *S*. *pneumoniae*, such as the synthetic promoter P3 (S2 Fig) [20]. The observed expression pattern allows for continuous expression of the regulatory genes, *blpSRH*, during all growth stages (Fig 3E). It should be noted that the P_*blpS*_ sequence (Fig 3B) is the least conserved of the promoters and that this sequence is located 100 bp upstream of the *blpS* start codon; thus there might be an unidentified, non-*blp* promoter that drives the seemingly constitutive expression of *blpSRH*. BlpSRH together with BlpC constitute a quorum sensing regulatory system, and we can therefore assume that the extracellular BlpC concentration required to activate BlpR is reached by the end of exponential phase. Accordingly, no expression could be observed from the regulated promoters (P_*blpT*_, P_*blpA*_, P_*pncW*_ and P_*blpK*_) when the regulatory genes *blpSRHC* were deleted (Fig 3D and 3E, S1 Fig). Furthermore, deletion of only *blpC* also abolished promoter activity, demonstrating that induction is mediated via BlpC (S1 Fig), and *blp* expression in this strain could be activated by addition of synthetic BlpC (S1 Fig). Although the expression dynamics from the four inducible promoters were the same, their expression strengths were variable; P_*blpK*_ was by far the strongest, showing approximately 3-fold higher maximum *luc* expression compared to P_*blpT*_, which again was stronger than P_*pncW*_ and P_*blpA*_ (Fig 3D, S1 Fig).

**Fig 4:**
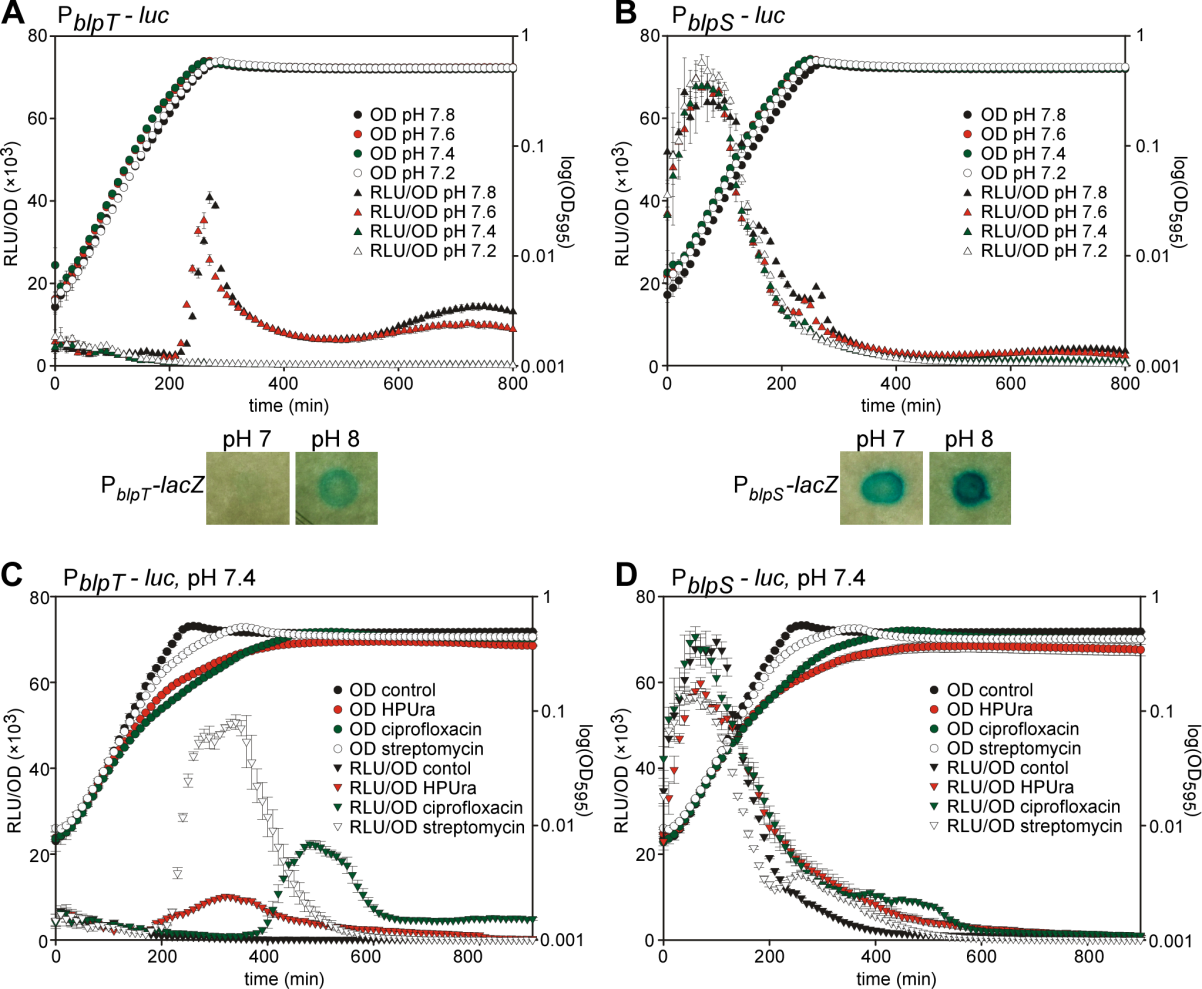
Conditions for induction of *blp* expression. (A) Expression from P_*blpT*_ is pH-dependent. Similar pH-dependent expression from promoters P_*blpA*_, P_*pncW*_ and P_*blpK*_ are shown in S3 Fig. (B) P_*blpS*_ activity is not affected by pH. For A and B, *S*. *pneumoniae* containing P_*blpT*_ and P_*blpS*_ reporter fusions, respectively, were grown in C+Y with different pH-values. LacZ production can be observed as blue colonies on plates. (C) Expression from P_*blpT*_ is induced by exposure to sub-lethal concentrations of competence-inducing antibiotics. Similar antibiotic-induced expression from promoters P_*blpA*_, P_*pncW*_ and P_*blpK*_ are shown in S4 Fig. (D) P_*blpS*_ activity is not significantly affected by antibiotic exposure. For C and D, *S*. *pneumoniae* containing P_*blpT*_ and P_*blpS*_ reporter fusions were grown in C+Y pH 7.4 with or without sub-lethal concentrations of antibiotics. HPUra (0.15 μg/ml), ciprofloxacin (0.4 μg/ml) and streptomycin (6 μg/ml) were added from the start of the growth curve. For all plots, gene expression as measured by luciferase activity (RLU/OD) is shown on the left axis and growth as measured by absorbance at 595 nm (OD_595_) is shown on the right axis. Averages of three replicates with the standard deviation are plotted.

By adjusting the pH of the growth medium, we observed that natural induction of all the regulated *blp* promoters in D39 only occurs above a threshold medium pH of 7.4 (Fig 4A and S3 Fig). pH-dependent expression is also seen in cells grown as colonies on agar plates (Fig 4A, bottom panel). Importantly, expression from P_*blpS*_ was independent of the initial pH of the medium (Fig 4B), confirming that this promoter is active across a broader range of environmental conditions.

To validate the RNA-seq results, which showed antibiotic-induced *blp* gene expression (S1 Table), we grew the reporter strains in C+Y pH 7.4 (which does not support *blp* induction) with and without sub-lethal concentrations of HPUra, ciprofloxacin and streptomycin. As shown in Fig 4C and S4 Fig, exposure to these competence-inducing antibiotics induced expression from all the regulated *blp* promoters (P_*blpT*_, P_*blpA*_, P_*pncW*_, P_*blpK*_). Importantly, exposure to rifampicin, which does not induce competence, also did not induce *blp* expression (S4 Fig). Moreover, the *blpS* promoter still displays activity throughout all growth stages, also when exposed to antibiotics (Fig 4D).

### Competence stimulating peptide (CSP) induces *blp* expression

**Fig 5:**
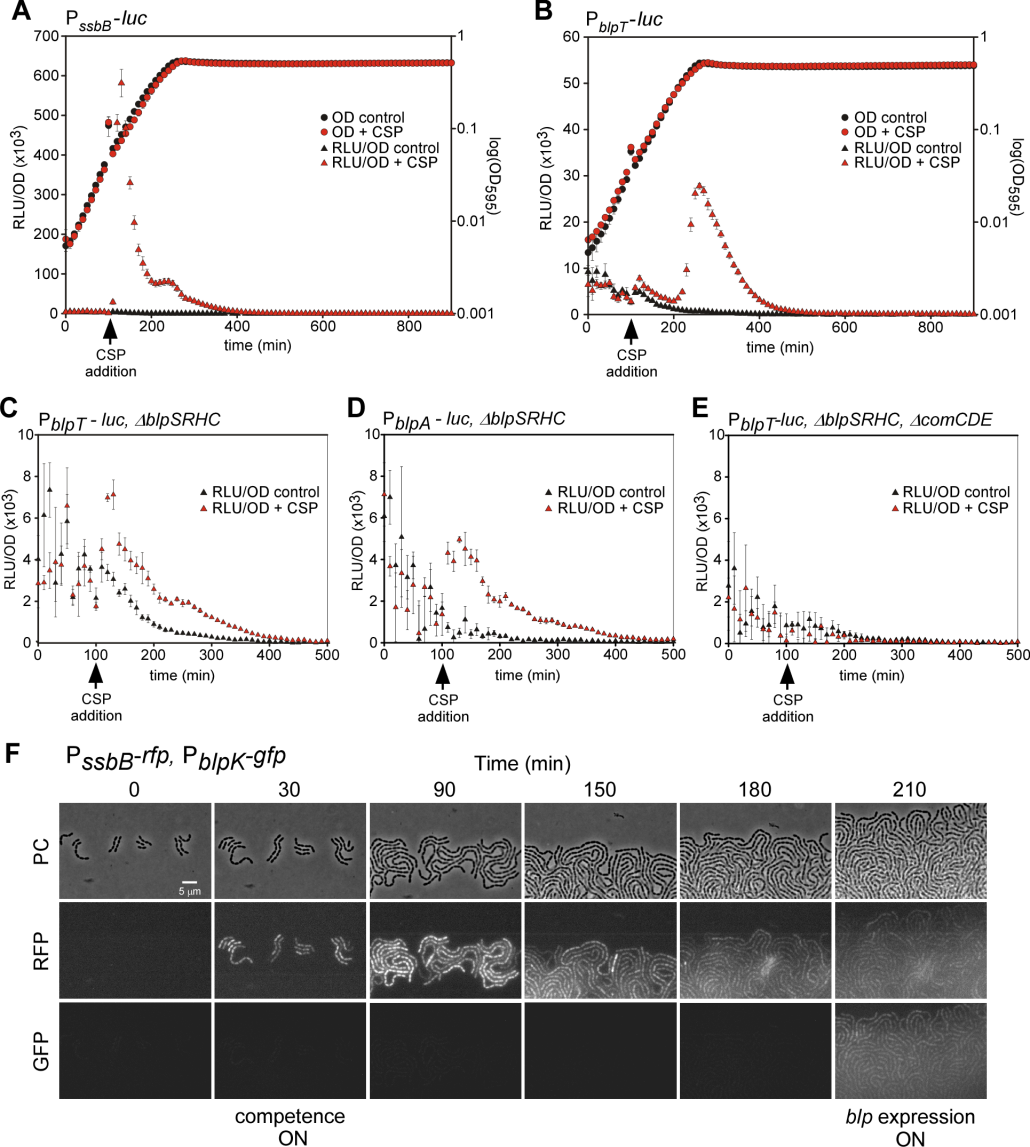
*blp* expression is induced by CSP. (A-B) Competence and *blp* expression are both stimulated by CSP addition, although with different kinetics. Competence reporter strain P_*ssbB*_-*luc* (A) and *blp* reporter strain P_*blpT*_-*luc* (B) grown in C+Y pH 7 with or without addition of 100 ng/ml CSP after 100 min (indicated by an arrow). Similar CSP induction of promoters P_*blpA*_, P_*pncW*_ and P_*blpK*_ are shown in S5 Fig. (C-D) CSP causes a weak but immediate activation of the *blp* promoters independent of *blpSRHC*. Reporter strains for P_*blpT*_ (C) and P_*blpA*_ (D), in which *blpSRHC* are deleted, grown in C+Y pH 7. (E) The immediate inducing effect is dependent on the ComCDE regulatory system. Expression from P_*blpT*_ in a strain with Δ*blpSRHC* and Δ*comCDE* grown in C+Y pH 7 is not induced by CSP addition. For extended plots of C-E, see S5 Fig. For all plots, gene expression as measured by luciferase activity (RLU/OD) is shown on the left axis and growth as measured by absorbance at 595 nm (OD_595_) is shown on the right axis. Averages of three replicates with the standard deviation are plotted. (F) *blp* expression is activated after competence in all cells. Time-lapse fluorescence microscopy of dual reporter strains for competence (P_*ssbB*_-*rfp*) and *blp* expression (P_*blpK*_-*gfp*) grown on C+Y agarose pH 8. PC; phase contrast.

The pH dependency we observed for the *blp* promoters (Fig 4A) was previously shown for the promoters of the competence regulatory system [15,16,21]. Furthermore, as described above, *blp* expression was induced by exposure to the same antibiotics that induce *com* expression (Fig 4C, S4 Fig, S1 Table). This prompted us to further investigate the putative interplay between the *blp* system and the *com* system. The *com* system can be induced by external addition of the competence stimulating peptide, CSP. By addition of CSP to cultures growing in C+Y pH 7, which normally does not allow for *blp* expression, we observed as expected that expression of the *com* gene *ssbB* was immediately induced [13,15] (Fig 5A). Crucially, addition of CSP to the *blp* reporter strains also led to high expression from all regulated *blp* promoters in the late exponential phase, approximately 100 min after addition of CSP (Fig 5B and S5 Fig). Notably, this delayed high induction of *blp* via CSP still depended on the *blp* regulatory system, as CSP-activated induction could not be observed when *blpSRHC* was deleted (S5 Fig).

The onset of high *blp* expression occurred 100 min after addition of CSP, prompting us to investigate in detail the more immediate consequences of exogenous CSP on *blp* promoters. We observed that addition of CSP caused a weak but consistent induction of the regulated *blp* promoters (Fig 5B–5D, S5 Fig). Importantly, the promoter controlling the inducer peptide *blpC*, P_*blpA*_, was also induced by CSP (Fig 5D). The same effects were observed in strains with or without *blpSRHC* deletion strains, which rule out any effect of binding by BlpR. The weak but immediate effects of the addition of CSP on the *blp* promoter suggest that the competence regulatory system (*comCDE*) can activate the *blp* promoters. To test this more specifically, we therefore deleted *comCDE*, and in this case, no direct effect or any delayed induction of *blp* expression was observed (Fig 5E, S5 Fig).

To further study the dynamics of *com*- and *blp* activation, we constructed a double reporter strain where (i) the *ssbB* promoter was transcriptionally fused to *mKate2*, a red fluorescent protein, and (ii) P_*blpK*_ was fused to *gfp*. Using fluorescence time-lapse microscopy under conditions that allowed natural *blp* and competence expression (pH 8.0), we observed that *blp* expression was activated after cells became competent (Fig 5F). This single cell analysis showed that all cells in the population turn on expression of *com* or *blp* synchronously once the systems are activated (Fig 5F).

### ComAB is responsible for processing/export of BlpC and thereby *blp* activation

**Fig 6:**
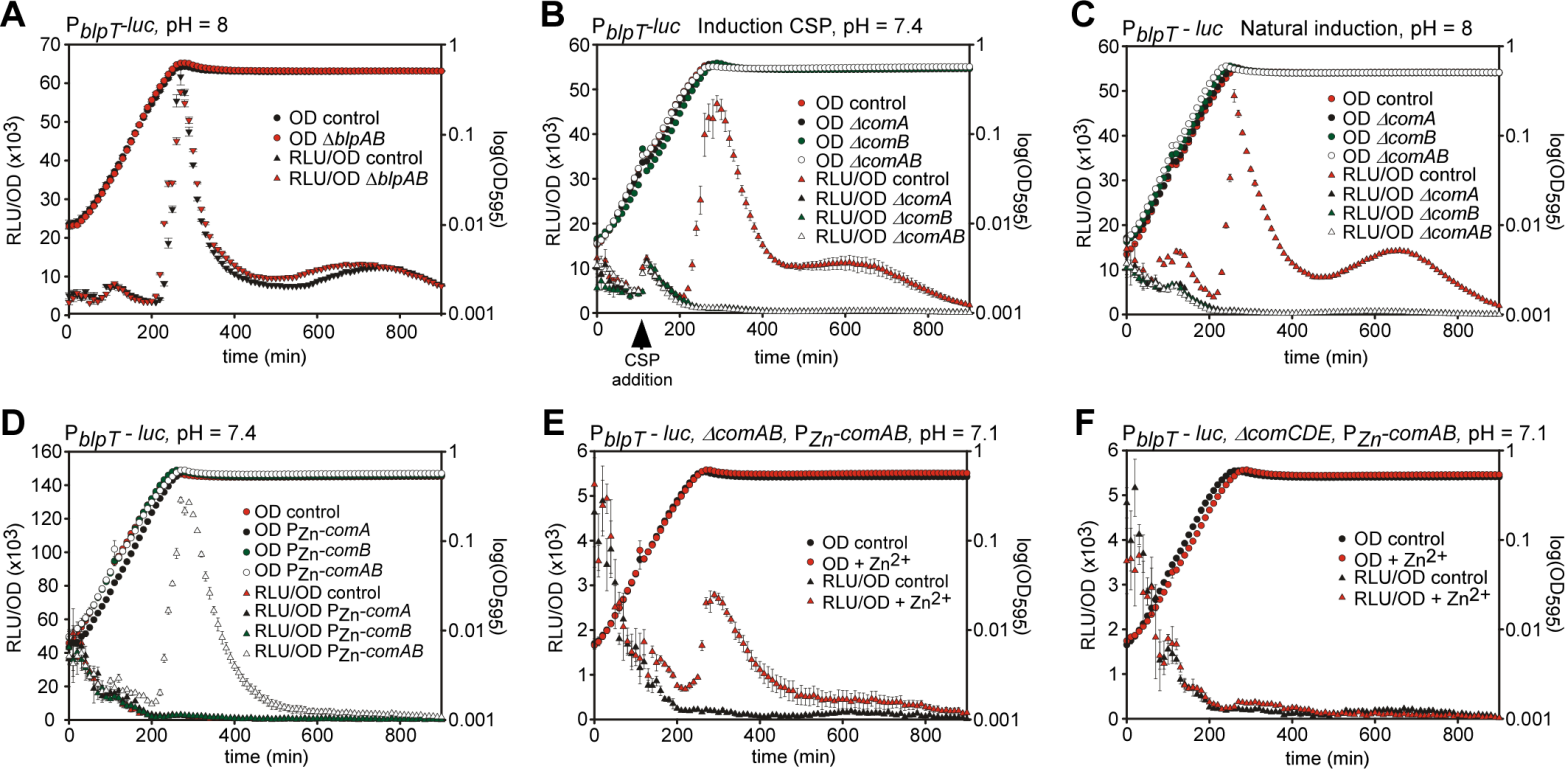
Interplay between *blp* and *com*. (A) The absence of *blpAB* does not abolish natural *blp* activation. Strains (wild type or Δ*blpAB*) carrying the P_*blpT*_ reporter were grown in C+Y pH 8 (similar results for P_*blpA*_, P_*pncW*_ and P_*blpK*_ in S6 Fig). (B) Induction of *blp* expression in late exponential phase by CSP can only occur if *comAB* is intact. Strains with the P_*blpT*_ reporter were grown in C+Y pH 7.4, and CSP (100 ng/ml) was added as indicated by the arrow. (C) Natural induction of *blp* expression can only occur if *comAB* is intact. Strains with the P_*blpT*_ reporter were grown in C+Y pH 8. (D) Ectopic, inducible expression of *comAB*, but not *comA* or *comB* alone, activate *blp* expression. Strains with the P_*blpT*_ reporter were grown in C+Y pH 7.4 with 0.1 mM ZnCl_2_ and 0.01 mM MnCl_2_ for induction. (E) Complementation of *blp* expression in a Δ*comAB* mutant by ectopic expression of *comAB* from a Zn-inducible promoter. (F) *comCDE* is required for *blp* activation in late exponential phase. Ectopic expression of *comAB* cannot activate *blp* expression when *comCDE* is absent suggesting that the weak activation of *blpC* transcription by *comE* is necessary for *blp* expression. For E and F, reporter strains were grown with or without 0.1 mM ZnCl_2_ and 0.01 mM MnCl_2_ in C+Y pH 7.1. For all plots, gene expression as measured by luciferase activity (RLU/OD) is shown on the left axis and growth as measured by absorbance at 595 nm (OD_595_) is shown on the right axis. Averages of three replicates with the standard deviation are plotted.

First, to investigate whether the frameshifted *blpAB* locus could still be responsible for export and processing of BlpC, we replaced the *blpAB* pseudogenes with an antibiotic resistance marker, while keeping the promoter and *blpC* intact. As shown in Fig 6A, the Δ*blpAB* strain did not change expression of *blp* from any of the promoters (Fig 6 and S6 Fig) thereby demonstrating that, under these conditions, the remnants of *blpAB* in strain D39 are not involved in BlpC processing and export.

Next, we tested if BlpC utilizes the ComAB complex for processing and export. ComAB is highly similar to BlpAB (protein sequences show 61.9% identity between ComA/BlpA, and 29.0% identity between ComB/BlpB) and is located downstream (within 2.2 kb) of the BlpR-regulated *blpK* gene. Our bioinformatics analysis showed that ComAB are highly conserved and intact in most pneumococcal strains (Fig 2). Indeed, when deleting *comAB*, or even *comA* or *comB* separately, *blp* expression could no longer be induced by CSP (Fig 6B) and, natural *blp* expression at pH 8.0 was also abolished (Fig 6C). Addition of extracellular BlpC, however, still induced *blp* expression in the *comAB* deletion strain (S7 Fig).

When expressing *comA*, *comB* or *comAB* from an inducible promoter at an ectopic locus, we observed that *comAB* (but not *comA* or *comB* alone) caused onset of *blp* expression independent of *blpAB*, but again this occurred in late exponential phase (Fig 6D and S7 Fig). Ectopic expression of *comAB* also induced *blp* expression when the native *comAB* genes were deleted, but to lower levels (Fig 6E). Finally, the *comAB*-mediated induction was dependent on *comCDE* (Fig 6F), showing that ComCDE-stimulated *blpC* expression is also essential for natural *blp* activation.

## Discussion

We and others recently discovered that exposing the human pathogen *S*. *pneumoniae* to antibiotics that either stall replication forks [16] or increase the misfolding of proteins [17] can induce competence, leading to increased transformation rates [15,16]. Here we show that exposing *S*. *pneumoniae* to the same types of antibiotics can also activate expression of the *blp* genes, encoding antimicrobial peptides as well as genes necessary for self-immunity, processing, transport and regulation. Because *blp* bacteriocins are involved in pneumococcal inter-strain competition [4], antibiotic exposure therefore has the potential to indirectly modify competitive interactions among coexisting pneumococcal strains within the human nasopharynx.

**Fig 7:**
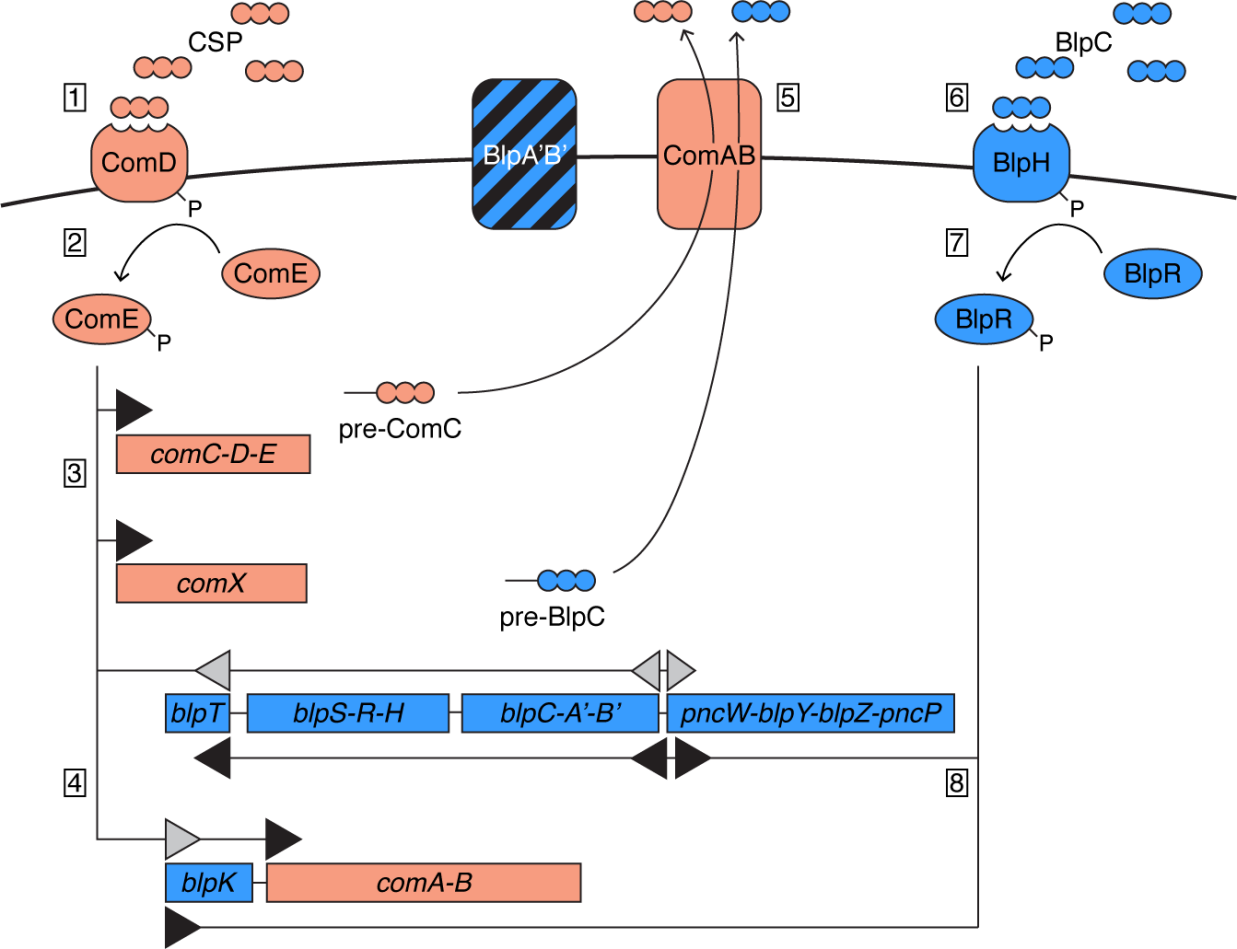
Model for competence-mediated *blp* activation. When the concentration of mature ComC (CSP) in the extracellular milieu reaches a threshold, CSP binds the membrane-located histidine kinase ComD (1), which autophosphorylates and transfers the phosphate group to activate the response regulator ComE (2). Activated ComE-P can bind to *com*-promoters to activate normal expression (3, black triangles), which includes *comC* and creates an autocatalytic loop. Expression of *comX*, which is an alternative sigma factor, leads to expression of the late competence genes encoding proteins involved in DNA transformation and repair. The current study suggests that activation of ComE-P might also directly induce expression of the *blp* promoters (4, grey triangles). This weak induction of BlpC production is necessary for triggering *blp* activation. BlpA’B’ is probably inoperative and pre-BlpC is instead processed and transported by ComAB (5). When the extracellular concentration of BlpC reaches a threshold, it binds to histidine kinase BlpH (6), which autophosphorylates and phosphorylates response regulator BlpR (7). Activated BlpR-P can then bind to and activate normal expression of the *blp* promoters (8, black triangles), which activate the genes encoding putative bacteriocins and their immunity proteins.

We show that regulation of *blp* expression is tightly linked with the competence regulatory system. *blp* and competence expression is regulated by paralogous quorum sensing based two-component systems. Over 75% of all pneumococcal strains sequenced to date (Fig 1) contain frameshift mutations in the ABC-transporter genes of the *blp* locus (i.e. *blpA* and *blpB*) thus rendering them inoperative and incapable of bacteriocin transport. Nevertheless, natural *blp* expression in strains with fragmented *blpAB* is frequently observed (Fig 3C, S1 Fig) [5,13,16], indicating that the inducer peptide BlpC as well as the bacteriocins, which are encoded with N-terminal double-glycine leader sequences, are processed and exported by other means. We show by deletion and complementation experiments that the competence transporter ComAB mediates processing and export of BlpC. However, expression of *comAB* alone is not sufficient to induce *blp* expression; because there is no (or very low) basal expression of *blpC* (Fig 5D and S1 Fig), a weak induction of *blpC* expression, probably via the competence response regulator ComE-P, is also required (Figs 5 and 6). Our study strongly suggests that the response regulator ComE-P can bind and activate the *blp* promoters. The response regulator binding boxes of the *blp* and *com* promoters are related, and there are even examples of promoters in *S*. *pneumoniae* which are activated by both ComE and BlpR [22], suggesting that such cross-induction can occur. The model for competence-dependent natural *blp* activation (Fig 7) thus involves at least two contact points between the two systems, and shows why the competence system needs to be activated before *blp* genes can be expressed. Moreover, this can explain why *blp* gene expression, in contrast to competence, is only observed in late exponential phase [6]. The gene encoding the competence induction peptide, *comC*, is co-transcribed with the regulatory genes which have a low basal expression level; therefore processing and export of CSP by the dedicated ComAB transporter can initiate in the early growth stages. By contrast, the gene encoding the *blp* induction peptide, *blpC*, is co-transcribed with the transporter genes *blpAB* and requires initial activation, probably via ComE-P, for basal expression. BlpC also needs to use the non-cognate transporter ComAB for processing and export (Fig 7), and this process may be inefficient compared to ComC export. Thus the accumulation of extracellular BlpC might be slow (or BlpH might require high levels of BlpC for autophosphorylation to occur), and this may explain the long delay between activation of competence and activation of *blp* expression (approximately 100 min). Another reason for slow BlpC accumulation may be that the number of active ComAB proteins present in cells is low after escape from the competent state. Furthermore, we cannot exclude that also other factors, for example unidentified σ^X^-dependent genes, are also involved in the late exponential phase activation of *blp* expression. Nevertheless, activation of *blp* expression is dependent on accumulation of two distinct quorum sensing peptides (CSP and BlpC), and this is, to the best of our knowledge, the first example of such a dual quorum sensing system. By hard-wiring bacteriocin expression with the competence system, expression is only activated at high cell densities when nutrients are scarce. Whether this timing is important for actual predation on competing bacteria, and how this occurs in strains with intact BlpAB remains to be determined. Furthermore, the mechanism responsible for shut-down of *blp*-expression as cultures enter stationary phase are also unknown, and future research should show whether competence genes are also involved in this process.

In a previous study, Son *et al*. [7] suggested that strains with interrupted *blpAB* are so-called cheater strains that are unable to secrete pneumocins and BlpC but still respond to peptides produced by coexisting strains. While cross-induction between strains may be possible via response to different allelic variants of secreted BlpC, our results clarify this is not required in interrupted *blpAB* strains; induction in these strains can be entirely due to endogenous BlpC production. We show that BlpC and possibly also pneumocins, can be exported via ComAB, suggesting that strains carrying BlpAB lesions are not cheaters. Importantly, however, autoinduction and cross-induction of *blp* expression within cells are not mutually exclusive and cross-induction can possibly occur under environmental conditions not allowing autoinduction. Many questions remain about the evolution of this apparently redundant BlpAB transport system. Our results suggest that once *blpA* frameshift mutations arise, deletions in *blpB* likely follow (Fig 1A). However, it is puzzling that the genes themselves are retained in most pneumococcal genomes despite the prediction that they would become further degraded by genetic drift since they are apparently not required for BlpC or bacteriocin transport. Our preliminary analysis suggests that *blpA* mutations have occurred independently several times during pneumococcal evolution (S8 Fig). Thus, an intriguing possibility is that our snapshot in time of this operon has captured the slow progression of the loss of this gene as its functions are overtaken by *comAB*. Alternatively, the kinetics of *blp* activation may be different in the minority (23.5%) of strains with intact *blpAB* alleles (Fig 1A), or (parts of) *blpAB* may retain functions in the export of bacteriocins under certain conditions, and this may further vary as a function of the position of the *blpA* frameshift. It is worth noting from Fig 1A that some parts of the *blpAB* genes (for example amino acid 1–159 encoding a peptidase domain) is retained in almost all strains. At the same time, since *blpC* is located on the same transcriptional unit as *blpAB*, it is possible that the operon is not further degraded to avoid interference with *blpC* transcription and translation.

During the competent state, pneumococci are able to lyse non-competent siblings in order to gain access to their DNA for natural transformation [19]. In contrast to the murein hydrolase CbpD and the CibAB bacteriocins, the Blp bacteriocins have not been implicated in fratricide, partially because their induction level upon competence activation by CSP appear low [19,23]. In the present work we show that although the initial *blp* activation in early exponential phase is indeed low, a strong induction of *blp* expression occurs approximately 100 minutes after competence, potentially causing high production of bacteriocins. The competence regulon is considered to consist of two sets of genes; those directly regulated by binding of ComE-P (early competence genes) and those that are activated by the alternative sigma factor, σ^X^, which is encoded by the ComE-P induced gene *comX* (late competence genes). Expression of both early and late genes occurs in the early exponential growth phase. The *blp* genes are also part of the competence regulon [13,16,23], but their dependence on the competence system is not via σ^X^ and also not solely via ComE-P, based on the mechanism described here (Fig 7). Therefore, our results clarify that the *blp* genes represents a separate subgroup within the competence regulon. Furthermore, since high level *blp* activation is competence-dependent and Blp bacteriocins have been shown to function in inter-strain competition [4], it remains possible that Blp-mediated cell lysis, together with CibAB and CbpD, can also contribute to the fratricide mechanism by releasing DNA from competitor strains [19]. One requirement for this proposed mechanism would be that competence is also activated. We observe in our *in vitro* experiments that the peak of bacteriocin export, and therefore potentially DNA release from dead cells, does not coincide with the peak of competence. This is the case both for natural expression and for antibiotic-induced expression of *com* and *blp*. Notably, however, it was recently shown that competence is constitutive in pneumococcal biofilms and *in vivo* [24], suggesting that pneumocin activity during competence is possible. However, further experimentation is required to test this hypothesis.

## Materials and Methods

### Bioinformatics

We complied 4,418 *S*. *pneumoniae* genomes from several data sets: 295 genomes from GenBank, which include 121 genomes from Georgia [25]; 3017 genomes from Myanmar refugees [26]; 616 genomes from Massachusetts [27]; 82 genomes from Complex 3 strains [28]; 240 genomes from PMEN-1 strains [29], 142 genomes from strains isolated from The Netherlands (European Nucleotide Archive study PRJEB10892) and 26 additional PMEN strains (European Nucleotide Archive study PRJEB10893). All sources collected strains without regard to strain identity, except for the Complex 3 and PMEN-1 research, which purposely focused on sampling subclades of *S*. *pneumoniae*. Therefore, excluding these two sources, we considered 4,096 of these genomes as randomly sampled from global populations.

We aligned the genomes to GenBank R6_uid57859 using Stampy 1.0.23 [30] with a substitution rate of 0.01. Based on the SNP data, we used FastTree 2.1.7 [31] with the ‘no maximum likelihood’ option, Jukes-Cantor nucleotide distances, and minimum 75% Shimodaira-Hasegawa local support to construct the full-genome phylogeny. We excluded any sites with more than 5% gaps. We included 69 *Streptococcus* sp. *viridans* as an outgroup clade for this phylogeny.

We used a reciprocal best-hit BLAST criterion to find sequences more similar to *blpA*, *blpB*, *comA*, and *comB* than other annotated *S*. *pneumoniae* genes. In this search, we only examined ORFs longer than 150 bp. We considered full-length alleles to be at least 717, 435, 717, and 449 residues long for each gene, respectively. *comA* and *comB* had 180 (34 over 0.5% proportion) and 121 (31 over 0.5% proportion) amino acid variants, respectively; we grouped the amino acid sequences by similarity as phylotypes. Amino acid sequences for each gene were aligned, and a neighbor-joining tree was created using Geneious 7.1.5 [32]. These gene trees were impartially divided into subtrees based on three restrictions: branches over 3.5 standard deviations in length from the mean branch length for the entire tree were cut; branches with length over 0.025 were cut; and clades were divided so the maximum intra-clade distance was 0.05. This lead to 5 phylotypes of *comA* over 0.5% proportion (A: 73.1%; B: 15.0%; C: 6.3%; D: 3.7%; E: 1.5%) and 2 phylotypes of *comB* over 0.5% proportion (A: 97.5%; B: 2.3%).

To find bacteriocins, we used a reciprocal best-hit BLAST criterion with annotated *blp* bacteriocins; we also examined all ORFs containing M[DN][TK]K leader sequence upstream of a GG site and all ORFs containing a ‘TMLS’ leader sequence upstream of a GG site. We classified any sequences with this feature that did not have a BLAST hit in the GenBank database as a bacteriocin. Afterwards, we found any resulting sequences mapped to either the *blp* locus or to the *comAB* locus, as expected of *blp* bacteriocins.

In order to test associations between gene types along the phylogeny, we calculated the maximum likelihood of two different models for each pair of gene lengths or phylotypes using BayesTraits 2.0 [33] and our phylogenetic tree. One model allowed the genes to mutate independently of each other; the other model had the mutation rate of each gene depend on the state of the other gene. We only considered gene length or phylotype pairs that co-occur in at least 0.5% of the 4,418 genomes. In all cases, strains with no allele of a gene were treated as missing data. We used a log ratio test between the two likelihood models to evaluate significance.

To reconstruct the phylogeny of the *blpA* region, a GTR+I+G model of evolution was employed, using Geneious 7.1.5 [32] and MrBayes 3.2.2 [34].

### Growth conditions and transformation

*S*. *pneumoniae* was grown in C+Y medium [35] at 37°C. For transformation, *S*. *pneumoniae* was grown until OD_600_ = 0.1 before cells were washed and incubated 12 min at 37°C with 100 ng/ml synthetic CSP-1. DNA to be transformed was added to the cells, followed by 20 min incubation at 30°C. Cells were then diluted 10 times in fresh medium and incubated for 1.5 hours at 37°C. The transformations were plated in Columbia agar supplemented with 2% (v/v) defibrinated sheep blood (Johnny Rottier, Kloosterzade, The Netherlands). For selection, the following concentrations of antibiotics were used: 1 μg/ml tetracycline, 100 μg/ml spectinomycin, 0.25 μg/ml erythromycin, 2 μg/ml chloramphenicol.

*Escherichia coli* was grown in LB at 37°C with shaking. *E*. *coli* was transformed with heat-shock of chemically competent cells according to standard protocols [36]. When appropriate, 100 μg/ml ampicillin or 100 μg/ml spectinomycin was used for selection.

### Strain construction

All strains and plasmids used in this study are listed in S2 Table. Oligonucleotides are listed in S3 Table.

### Construction of triple reporter cassette

A reporter cassette containing three reporter genes, firefly luciferase (*luc*), superfolder GFP, (*(sf)gfp*), and β-galactosidase (*lacZ*) was amplified from plasmid pAD4 (A. Domenech and J.-W. Veening) using primers OG48 and OG50. The fragments were digested with restriction enzymes AseI and BamHI and ligated into the corresponding site of plasmid pPEP1 [20], a vector which replicates in *E*. *coli* and integrates into the *cep*-locus of *S*. *pneumoniae* D39 by double crossover. The resulting reporter plasmid (Fig 3C), suitable for insertion of promoter fragments upstream of the triple reporter system, was called pPEP1-LGZ.

### Construction of promoter fusions to reporter cassette

For construction of pPEP1-P_*blpT*_-LGZ, the promoter fragment P_*blpT*_ was amplified from genomic DNA of *S*. *pneumoniae* D39 using primers OG56 and OG26. The fragment was digested with enzymes NheI and BglII and ligated into the corresponding restriction sites of plasmid pPEP1-LGZ. Using the same template DNA, promoter fragments P_*blpK*_ was amplified with primers Pspd_0046-F+NheI+NotI and Pspd_0046-R+BglII, P_*blpS*_ with primers PblpS_F_NheI_NotI and PblpS_R_BglII, P_*blpA*_ with primers PblpA_F_NheI_NotI and PblpA_R_BglII and P_*pncW*_ with primers PblpU_F_NotI_NheI and PblpU-R+BamHI. For all promoter fragments, we amplified a region of 250 bp, containing 200 bp upstream and 50 bp downstream of the startcodon of the first gene in each transcriptional unit. Fragments were ligated into pPEP1-P_*blpT*_-LGZ using restriction sites NheI and BglII to construct vectors pPEP1-P_*blpK*_-LGZ, pPEP1-P_*blpS*_-LGZ, pPEP1-P_*blpA*_-LGZ and pPEP1-P_*pncW*_-LGZ, respectively. The ligations were transformed into *E*. *coli* DH5a and transformants were selected with 100 μg/ml spectinomycin. Correct plasmids were verified by sequencing and transformed into *S*. *pneumoniae* D39. Correct integration of the plasmids via double crossover in the pneumococcal genome was verified by PCR.

### Deletion of *blpSRHC*

The regulatory genes of the *blp* operon, *blpSRHC*, were deleted using allelic replacement with an erythromycin resistance cassette. The erythromycin resistance cassette was amplified from strain MK110 [16], using primers eryR-up_F_BamHI and eryR-down+Not. The region upstream of *blpS* was amplified using primers Blp_SRHC_up_F and Blp_SRHC_up_R_BamHI, while the region downstream of *blpC* was amplified with primers Blp_SRHC_dn_F_NotI and Blp_SRHC_dn_R. The upstream fragment was digested with BamHI, the downstream fragments with NotI and the fragments containing the erythromycin cassette with both BamHI and NotI. The three fragments were ligated and transformed into *S*. *pneumoniae* D39 using 0.25 μg/ml erythromycin for selection. Correct deletion of *blpSRHC* was confirmed by PCR and sequencing.

### Deletion of *bgaA*

The *S*. *pneumoniae* β-galactosidase gene *bgaA* was deleted by transforming the plasmid pMK11 (see below), containing a tetracycline resistance gene and the P_*Zn*_ promoter, into *S*. *pneumoniae*. Transformants were selected using 1 μg/ml tetracycline.

### Deletion of *comA*, *comB* and *comAB*

*comA*, *comB* and *comAB* were deleted by allelic replacement with an erythromycin resistance cassette. Briefly, the region upstream of *comA* was amplified with primers comA1 and comA2+AscI and the region downstream of *comA* was amplified with comA3+NotI and comA4. Genomic DNA from *S*. *pneumoniae* D39 was used as template. The erythromycin resistance cassette was amplified from genomic DNA of strain *ΔhexA*::*ery* [37] using primers trmp-F+AscI and sPG20_eryR+NotI. The *comA*-up fragment was digested with restriction enzyme AscI, the erythromycin resistance cassette fragment with AscI and NotI and the *comA*-down fragment with NotI. The three fragments were ligated and transformed into *S*. *pneumoniae*. Transformants were selected with 0.25 μg/ml erythromycin and correct transformants were verified by PCR and sequencing.

*comB* was deleted in a similar fashion. The *comB*-up fragment was amplified using primers comB_up_R+AscI and ComA1, the *comB*-down fragment with primers comB_down_F+NotI and comB_down_R, and the *ery* fragment was amplified using primers trmp_F+Ascl and SPG20_eryR+NotI with *ΔcomA*::*ery* strain as a template. For deletion of *comAB*, a fragment containing the *comAB*-up region and the erythromycin resistance gene was amplified with primers comA1 and SPG20_eryR+NotI using the *ΔcomA*::*ery* strain as a template. The *comAB*-down fragment was amplified using primers pr225comB_down_F+NotI and pr226comB_down_R. Genomic DNA was used as template in all cases. Digestion, ligation and transformation was performed in the same manner as for the Δ*comA*::*ery* deletion, to generate strains Δ*comB*::*ery* and Δ*comAB*::*ery*.

### Deletion of *comCDE*

The competence regulatory operon *comCDE* was deleted with allelic replacement with an chloramphenicol resistance cassette, as described previously [16].

### Construction of pMK11, a plasmid for Zn-inducible expression

A Zn^2+^-inducible promoter was amplified from plasmid pJWV25 [38] using primers pr27 and pr28. The fragment was digested with SphI and SpeI and ligated into the corresponding sites in plasmid pJWV100 [39] to create pMK11.

### Constructs for inducible expression of *comA*, *comB* and *comAB*

For controlled expression of *comA*, *comB* or *comAB*, the gene(s) were inserted downstream of a Zn^2+^ inducible promoter and integrated in the genome at the *bgaA*-locus. *comA* was amplified using primers start-comA+EcoRI and end-comA+SpeI, *comB* with primers start-comB+EcoRI and end-comB+SpeI and *comAB* with primers start-comA+EcoRI and end-comB+SpeI. The fragments were digested with restriction enzymes EcoRI and SpeI/BcuI, and ligated into the corresponding sites of plasmid pMK1, to generate plasmids pMK11-P_Zn_-*comA*, pMK11-P_Zn_-*comB* and pMK11-P_Zn_-*comAB*. The ligation was transformed into *E*. *coli* using ampicillin selection (100 μg/ml). Correct plasmids were verified by sequencing and then transformed in *S*. *pneumoniae* using tetracycline selection (1 μg/ml).

### Deletion of *blpAB* pseudogenes

The *blpAB* pseudogenes in *S*. *pneumoniae* D39 were deleted by replacement with an erythromycin resistance cassette. The promoter P_*blpA*_ and *blpC* were kept intact. The region downstream of *blpAB* was amplified using primers blpB-down-F and blpB-down-R-NotI, while the region upstream of *blpAB* was amplified with primers blpA-up-F-BamHI and blpA-up-R. In both cases, genomic DNA from *S*. *pneumoniae* D39 was used as template. The erythromycin resistance gene was amplified from strain MK304 using primers Ery-For-BamHI and sPG20_eryR+NotI. The up- and downstream fragments were digested with BamHI and NotI, respectively, while the fragment containing the erythromycin resistance gene was digested with both BamHI and NotI. The fragments were ligated and the construct was transformed into *S*. *pneumoniae* D39. In all steps of the transformation reaction, 500 ng/ml CSP-1 was added to induce expression from the P_*blpA*_ promoter driving expression of the erythromycin resistance gene. Cells were plated on C+Y agar plates containing erythromycin for selection and 500 ng/ml CSP-1. The construct in the resulting strain was confirmed by PCR and sequencing.

### Construction of double-labeled reporter strain P_*ssbB*_-*rfp* and P_*blpK*_-*gfp* for time-lapse microscopy

D39 was transformed with plasmid pPEP1-P_*blpK*_-LGZ (including the *gfp* reporter), which integrates in the *cep*-locus. Transformants were selected on plates with 100 μg/ml spectinomycin. This new strain was subsequently transformed with plasmid pLA21 [37] containing the P_*ssbB*_-*rfp* fusion which integrates in the *bgaA*-locus. In the second transformation round, colonies were selected on 1 μg/ml tetracycline.

### RNA-sequencing

For RNA-sequencing, samples of *S*. *pneumoniae*, strain DLA3 (*bgaA*::P_*ssbB*_-*luc*) were grown to OD_600_ = 0.4 in 5 ml tubes and diluted 1:100 in fresh C+Y medium (pH 7.4). To study the effects of antibiotics, cells grown without antibiotics were compared to cells grown with 0.4 μg/ml ciprofloxacin, 608 μg/ml hydroxyurea, or 0.04 μg/ml rifampicin. Cells were grown in microtiter plates and growth and competence development (*luc*-expression) were followed. For all the samples, when one-third of the maximum OD_600_ was reached, cells were harvested by centrifugation (7,500 rcf for 5 min) and frozen. For RNA isolation, cells were lysed by bead beating and RNA was purified using phenol-chloroform extractions and ethanol precipitations. DNA was removed from the sample with RNase-free DNase I (Fermentas) treatment for 45 min. Ribolock (Fermentas) was added to avoid RNA degradation.

Library preparation and whole-genome sequencing were performed by vertis Biotechnologie AG (Freising, Germany). Ribosomal RNA was removed using the Ribo-Zero rRNA Removal Kit (Epicenter) prior to preparation of cDNA libraries. Sequencing of the cDNA libraries was performed with an Illumina HiSeq 2000 machine with 100 nt read length paired end.

Sequence reads were mapped to the *S*. *pneumoniae* D39 genome (NC_008533) using Rockhopper version 2.03 [40,41], using default parameters. Reads Per Kilobase of exon per Megabase of library size (RPKM) were calculated using a protocol from Chepelev *et al*. [42]. In short, exons from all isoforms of a gene were merged to create one meta-transcript. The number of reads falling in the exons of this meta-transcript were counted and normalized by the size of the meta-transcript and by the size of the library. This was done internally by Rockhopper version 2.03 after aligning reads. Upper quartile normalization (to be able to compare expression between samples) was used to transform RPKM (reads per kilo base per million) values into expression values, performed by Rockhopper. Finally, Rockhopper was also used for differential gene expression analysis, using default parameters.

Sequencing data used in this paper have been deposited in the Gene Expression Omnibus repository (http://www.ncbi.nlm.nih.gov/geo/) with accession numbers GSE54199 and GSE69729.

### Luminescence assays

*S*. *pneumoniae* were pre-grown to OD_600_ 0.4 and diluted 100-fold in C+Y medium containing 340 μg/ml luciferin prior to the assay. pH of the medium was adjusted with HCl or NaOH. Production of firefly luciferase (encoded by *luc*) cause emission of light when the medium contains luciferin [43]. Luminescence assays were performed in 96-wells plates at 37°C. Absorbance (OD_595_) and luminescence (as relative luminescence units, RLU) were measured every 10 min for at least 13 hours using a Tecan Infinite 200 PRO instrument. When appropriate, 100 ng/ml synthetic peptide CSP-1 or 500 ng/ml BlpC purchased from Genscript (Piscataway, NJ) were added to the plates after 100 min. Concentrations were selected based on previous studies [9,16].

### LacZ reporter assay

β-galactosidase activity was assayed on agar plates. X-gal (40 μl of 40 mg/ml stock solution) was added on top of C+Y agar with adjusted pH. Twenty μl of *S*. *pneumoniae* at OD_600_ = 0.4 (approx. 10^7^ cells/μl) was then spotted. The drops with bacterial culture were allowed to dry prior to incubation overnight in a 5% CO_2_ incubator at 37°C.

### Time-lapse fluorescence microscopy

Time-lapse fluorescence microscopy was performed using a Deltavision Elite (GE Healthcare, USA) as described before [44,45]. In short, cells were grown until OD_600_ = 0.08 before they were spotted onto C+Y (pH 8) agarose slides. The slides were kept in a temperature controlled chamber at 37°C, and images (GFP, RFP and phase contrast) were acquired with a sCMOS camera every 10 min.

## Supporting Information

S1 Fig Natural induction of *blp* promoters. Reporter strains for *blp* promoters (A) P_*blpA*_, (B) P_*pncW*_ and (C) P_*blpK*_ grown in C+Y pH 8. (D) Strains containing the reporter P_*blpT*_ grown in C+Y pH 8. When *blpC* is deleted, no natural induction is observed, however by external addition of BlpC (timing indicated by arrow), *blp* expression is immediately switched on. For all plots, gene expression as measured by luciferase activity (RLU/OD) is shown on the left axis and growth as measured by absorbance at 595 nm (OD_595_) is shown on the right axis. Averages of three replicates with the standard deviation are plotted.
(TIF)

S2 Fig Comparison of expression dynamics of the synthetic, constitutive promoter P3 and promoter P_*blpS*_. Strains harboring the reporter construct P3-luc (A) or P_*blpS*_-luc (B) were pre-grown to OD_600_ = 0.4 and inoculated at four different initial cell densities (10^−2^ in black, 10^−3^ in red, 10^−4^ in green and 10^−5^ in blue) in C+Y pH 8. Growth curves (OD_595_, upper panels) and gene expression (RLU/OD, lower panels) over time are shown. The expression dynamics from the two promoters appear similar; expression starts when cells enter the exponential growth phase and the promoters remain active until stationary phase. Averages of three replicates with the standard deviation are plotted.
(TIF)

S3 Fig pH-dependent *blp* expression. Strains containing the promoter reporter fusions for P_*blpA*_ (A), P_*pncW*_ (B) and P_*blpK*_ (C) were grown in C+Y with different initial pH. Natural induction for all promoters is only observed for pH > 7.4. Gene expression as measured by luciferase activity (RLU/OD) is shown on the left axis and growth as measured by absorbance at 595 nm (OD_595_) is shown on the right axis. Averages of three replicates with the standard deviation are plotted.
(TIF)

S4 Fig Antibiotic-induced *blp* expression. *blp* reporter strain for P_*blpA*_ (A), P_*pncW*_ (B) and P_*blpK*_ (C) grown with or without sub-lethal concentrations of HPUra (0.15 μg/ml), ciprofloxacin (0.4 μg/ml) or streptomycin (6 μg/ml) shows that competence-inducing antibiotics also induce *blp* expression. (D) When P_*blpK*_ was grown with sub-lethal concentrations of rifampicin (0.04 μg/ml), which does not induce competence, *blp* expression was also not induced. For all plots, cells were grown in C+Y pH 7.4. Gene expression as measured by luciferase activity (RLU/OD) is shown on the left axis and growth as measured by absorbance at 595 nm (OD_595_) is shown on the right axis. Averages of three replicates with the standard deviation are plotted.
(TIF)

S5 Fig Effect of CSP on *blp* expression. CSP induces expression from regulated *blp* promoters P_*blpA*_ (A), P_*pncW*_ (B) and P_*blpK*_ (C), but does not affect expression from P_*blpS*_ (D). Upon addition of CSP an immediate (weak) induction of the promoter fusions in A-C were observed, while a delayed full activation of these promoters were observed in late exponential phase. The immediate activation is independent of *blpSRHC*, but the delayed full activation is dependent of *blpSRHC*, since reporter strains for P_*blpT*_ (E) or P_*blpA*_ (F) with deleted *blp* regulatory genes (*ΔblpSRHC*) still show similar levels of immediate induction, but no delayed induction (compare Fig 5B with panel E and panels A with F). (G) No CSP response is observed for P_*blpT*_ when *blpSRHC* and *comCDE* are deleted. For all plots, strains were grown in C+Y pH 7 with or without addition of CSP after 100 min (indicated by an arrow). Gene expression as measured by luciferase activity (RLU/OD) is shown on the left axis and growth as measured by absorbance at 595 nm (OD_595_) is shown on the right axis. Averages of three replicates with the standard deviation are plotted.
(TIF)

S6 Fig Deletion of *blpAB* pseudogenes is not detrimental for natural *blp* expression. Reporter strains for the promoters P_*blpA*_ (A), P_*pncW*_ (B), P_*blpK*_ (C) and P_*blpS*_ (D) with and without deleted *blpAB* pseudogenes show no differences in activity when grown in C+Y pH 8. Gene expression as measured by luciferase activity (RLU/OD) is shown on the left axis and growth as measured by absorbance at 595 nm (OD_595_) is shown on the right axis. Averages of three replicates with the standard deviation are plotted.
(TIF)

S7 Fig Induction of *blp* expression by external BlpC or over-expression of *comAB*. (A) *blp* expression can still be induced by external addition of BlpC in a *comAB* deletion strain. The P_*blpT*_ reporter strain with deleted *comAB* was grown in C+Y pH 7.1. BlpC was added after 100 min, as indicated by the arrow. (B) Overexpression of *comAB* induces *blp* expression also in a *blpAB* deletion strain. The P_*blpT*_ reporter strain with Zn^2+^-inducible *comAB*-expression was grown in C+Y pH 7.4 with or without the presence of 0.1 mM ZnCl_2_ and 0.01 mM MnCl_2_ for induction. Gene expression as measured by luciferase activity (RLU/OD) is shown on the left axis and growth as measured by absorbance at 595 nm (OD_595_) is shown on the right axis. Averages of three replicates with the standard deviation are plotted.
(TIF)

S8 Fig Phylogeny of the *blpA* region. We aligned the nucleotides of the *blpA* locus regardless of the presence of ORFs with 4 alleles of *comA* as an outgroup. Sites with more than 5% gaps were removed. Using a GTR+I+G model of evolution, we used Geneious 7.1.5 and MrBayes 3.2.2 to reconstruct the phylogeny of *blpA*. We collapsed clades with a posterior probability of less than 0.95. In red are two clades with full-length *blpA* alleles as the parsimonious ancestor that contain derived interrupted *blpA* alleles. Alleles coding for full-length *blpA* are in black circles; alleles producing interrupted *blpA* are colored by the length classes found in Fig 1A. Alleles producing interrupted *blpA* with the 1–195 fragment as in Fig 1A are shown as half-colored circles.
(TIF)

S1 Table Transcriptional response of *blp* genes of *S*. *pneumoniae* D39 after exposure to sub-lethal level of antibiotics as determined by RNA sequencing. (DOCX)

S2 Table Strains and plasmids used in this study. (DOCX)

S3 Table Oligonucleotides used in this study. (DOCX)
