# Supplementary figures and images for: Expression of Streptococcus pneumoniae Bacteriocins Is Induced by Antibiotics via Regulatory Interplay with the Competence System
Source: PLoS Pathog. 2016 Feb 3;12(2):e1005422. doi: 10.1371/journal.ppat.1005422 (PMC4739728; doi:10.1371/journal.ppat.1005422)

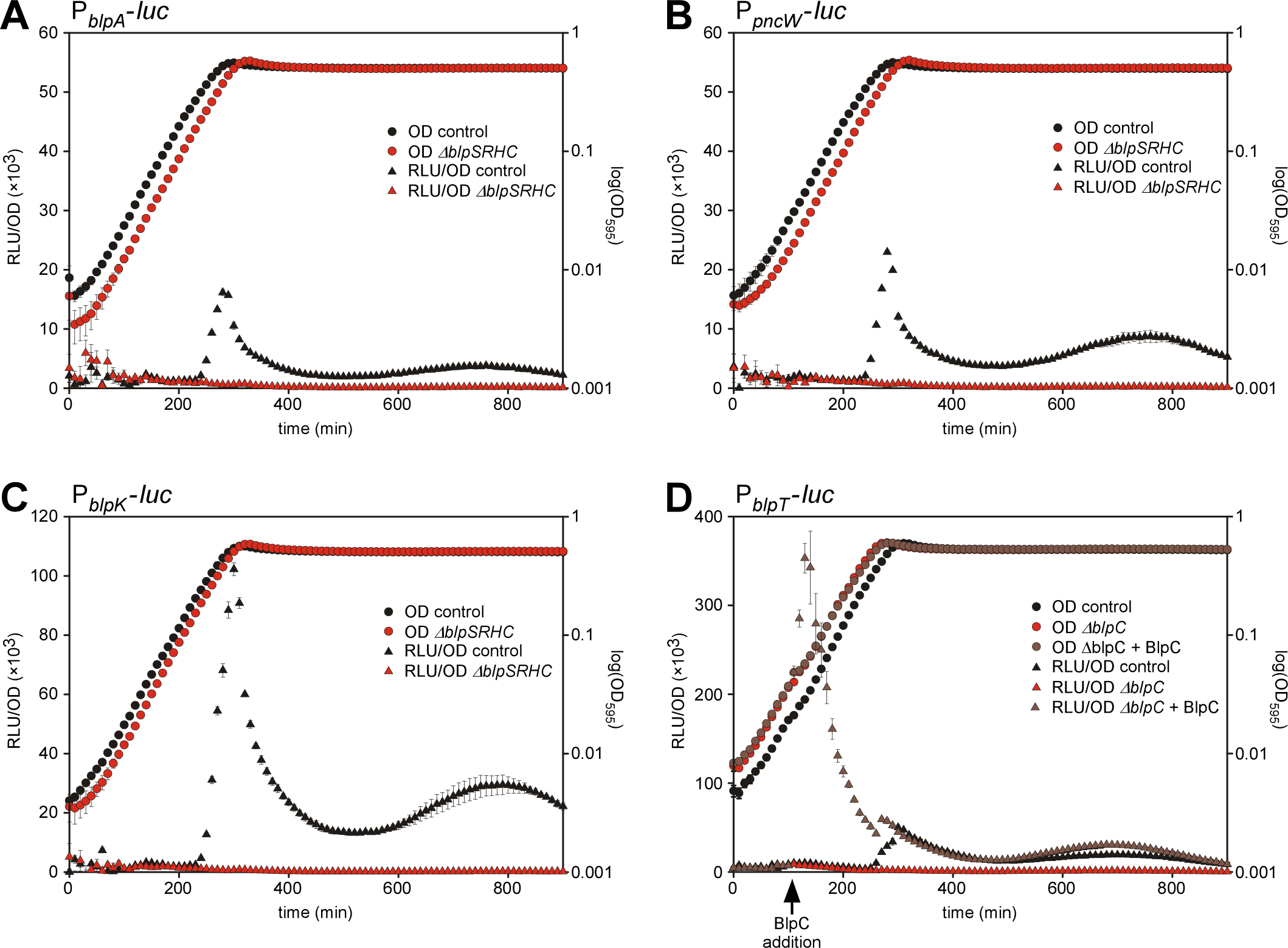

Supplement: S1 Fig — Reporter strains for blp promoters (A) PblpA, (B) PpncW and (C) PblpK grown in C+Y pH 8. (D) Strains containing the reporter PblpT grown in C+Y pH 8. When blpC is deleted, no natural induction is observed, however by external addition of BlpC (timing indicated by arrow), blp expression is immediately switched on. For all plots, gene expression as measured by luciferase activity (RLU/OD) is shown on the left axis and growth as measured by absorbance at 595 nm (OD595) is shown on the right axis. Averages of three replicates with the standard deviation are plotted. (TIF) [file ppat.1005422.s001.tif]

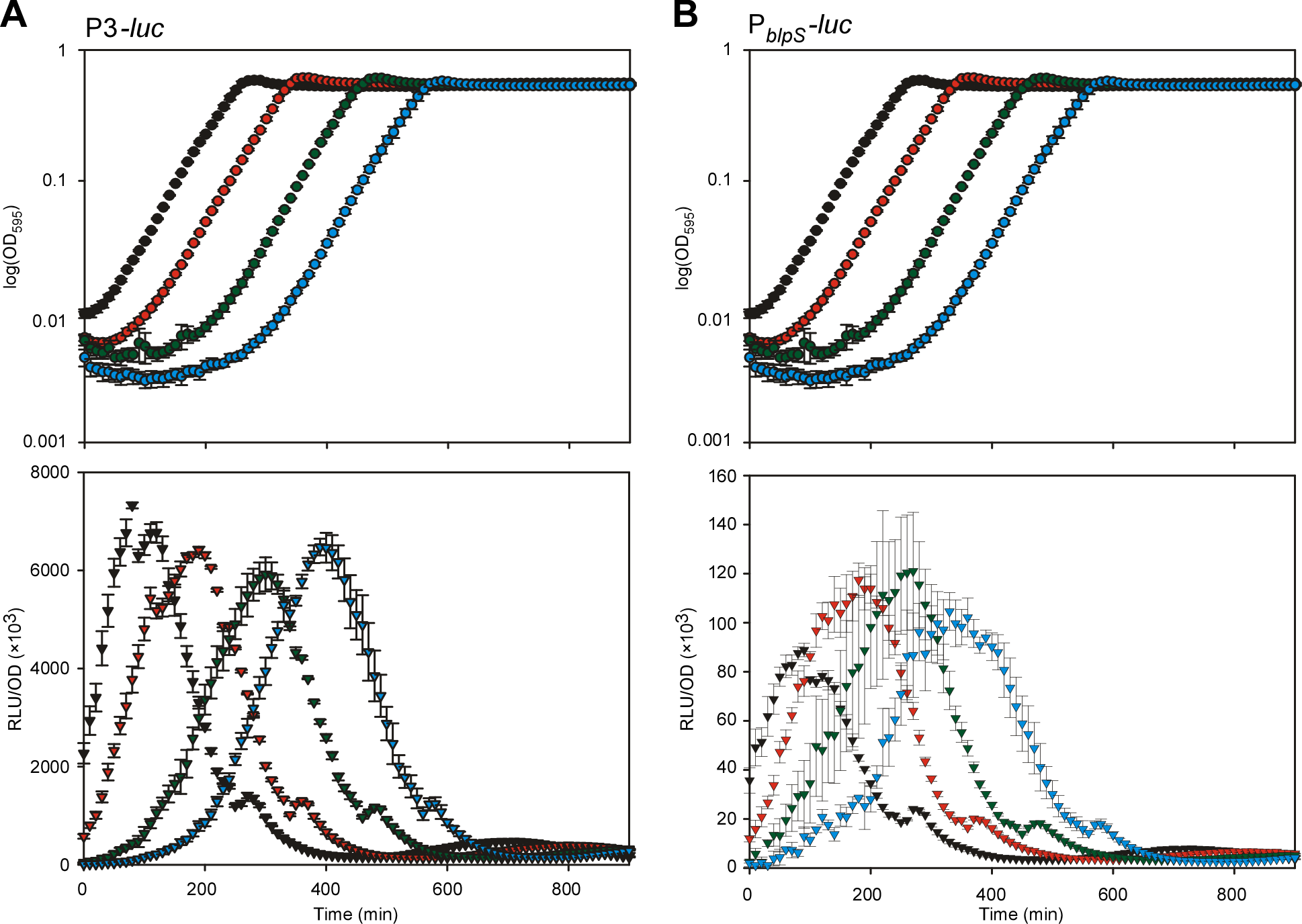

Supplement: S2 Fig — Strains harboring the reporter construct P3-luc (A) or PblpS-luc (B) were pre-grown to OD600 = 0.4 and inoculated at four different initial cell densities (10−2 in black, 10−3 in red, 10−4 in green and 10−5 in blue) in C+Y pH 8. Growth curves (OD595, upper panels) and gene expression (RLU/OD, lower panels) over time are shown. The expression dynamics from the two promoters appear similar; expression starts when cells enter the exponential growth phase and the promoters remain active until stationary phase. Averages of three replicates with the standard deviation are plotted. (TIF) [file ppat.1005422.s002.tif]

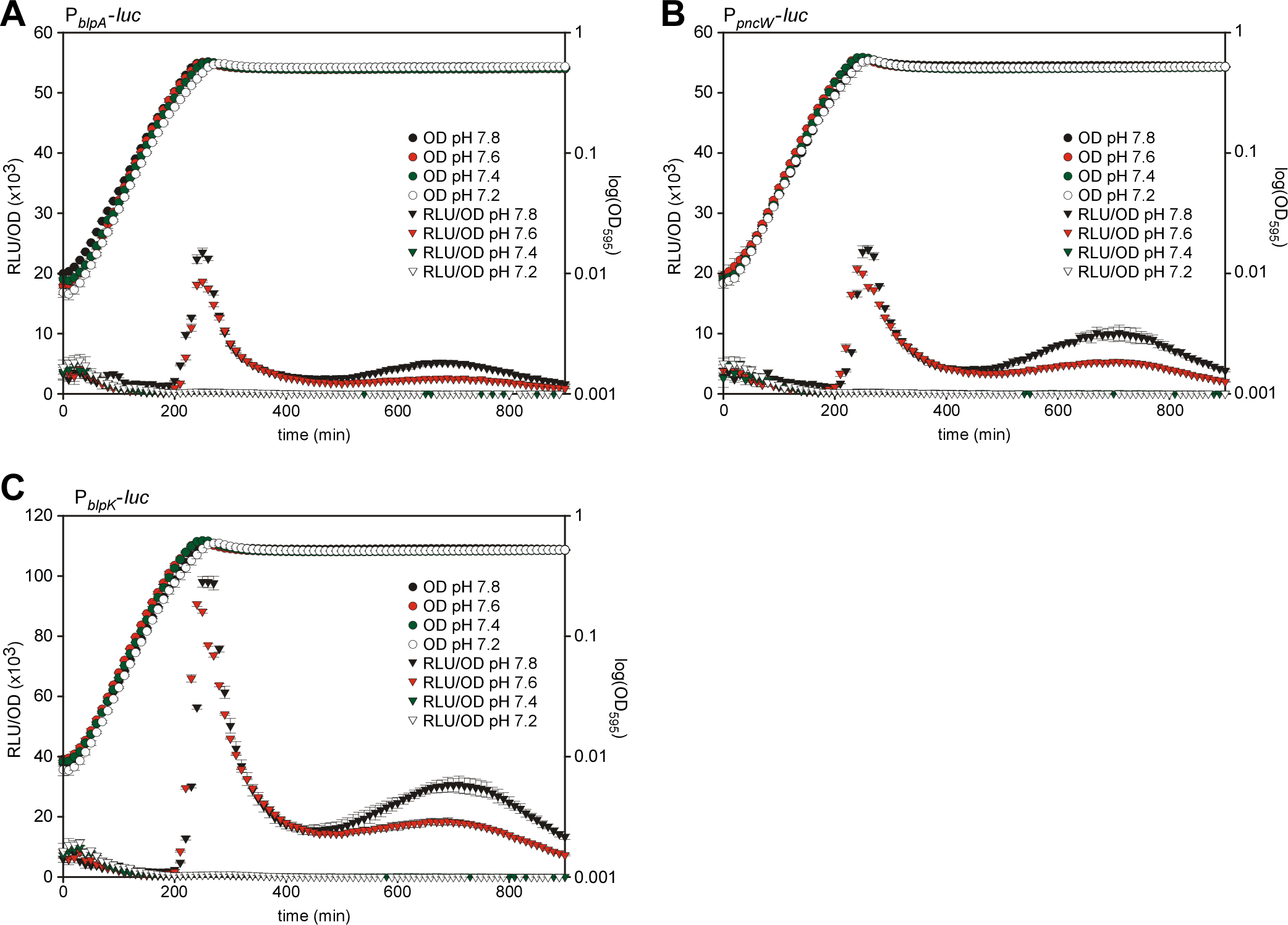

Supplement: S3 Fig — Strains containing the promoter reporter fusions for PblpA (A), PpncW (B) and PblpK (C) were grown in C+Y with different initial pH. Natural induction for all promoters is only observed for pH > 7.4. Gene expression as measured by luciferase activity (RLU/OD) is shown on the left axis and growth as measured by absorbance at 595 nm (OD595) is shown on the right axis. Averages of three replicates with the standard deviation are plotted. (TIF) [file ppat.1005422.s003.tif]

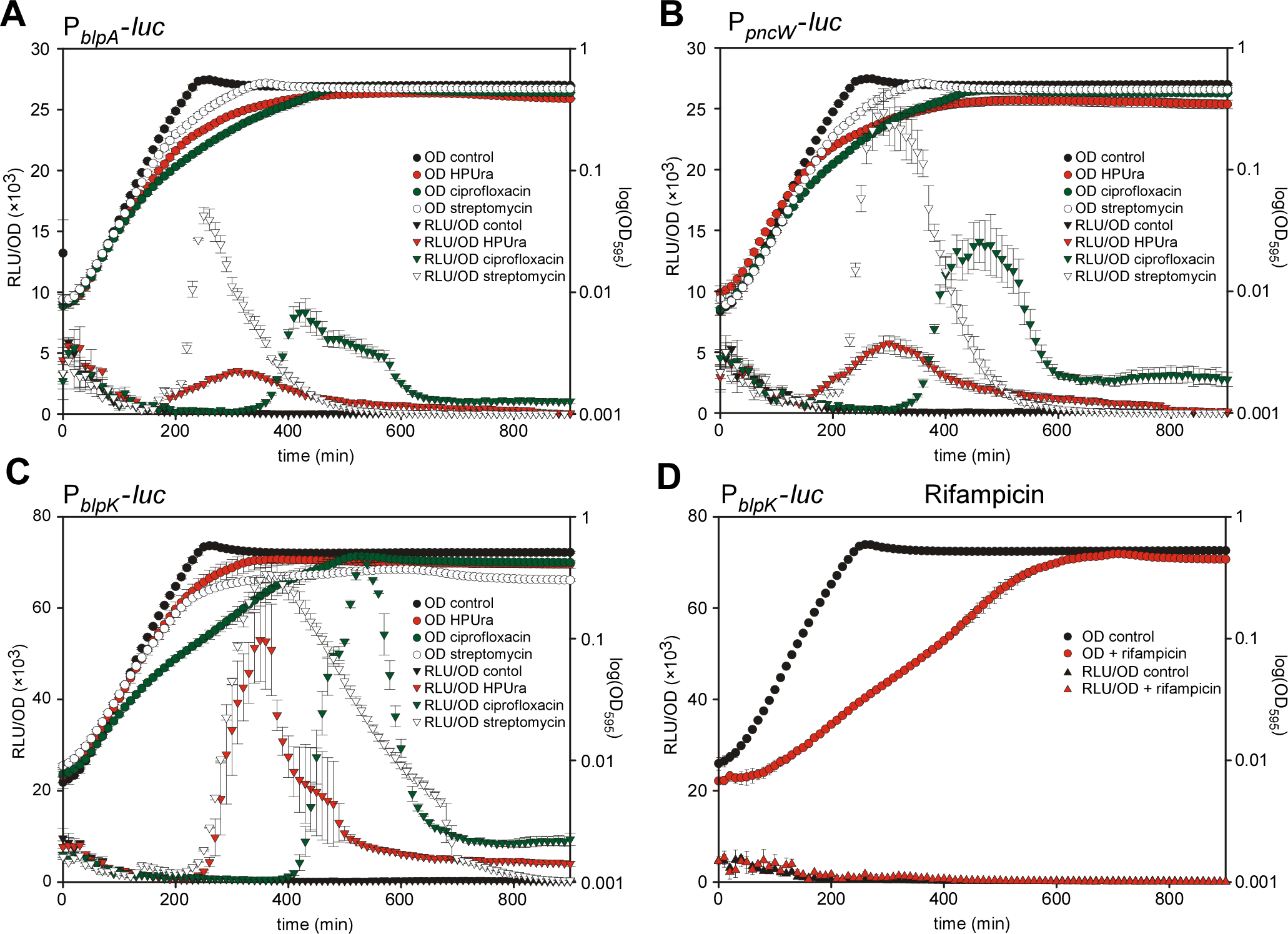

Supplement: S4 Fig — blp reporter strain for PblpA (A), PpncW (B) and PblpK (C) grown with or without sub-lethal concentrations of HPUra (0.15 μg/ml), ciprofloxacin (0.4 μg/ml) or streptomycin (6 μg/ml) shows that competence-inducing antibiotics also induce blp expression. (D) When PblpK was grown with sub-lethal concentrations of rifampicin (0.04 μg/ml), which does not induce competence, blp expression was also not induced. For all plots, cells were grown in C+Y pH 7.4. Gene expression as measured by luciferase activity (RLU/OD) is shown on the left axis and growth as measured by absorbance at 595 nm (OD595) is shown on the right axis. Averages of three replicates with the standard deviation are plotted. (TIF) [file ppat.1005422.s004.tif]

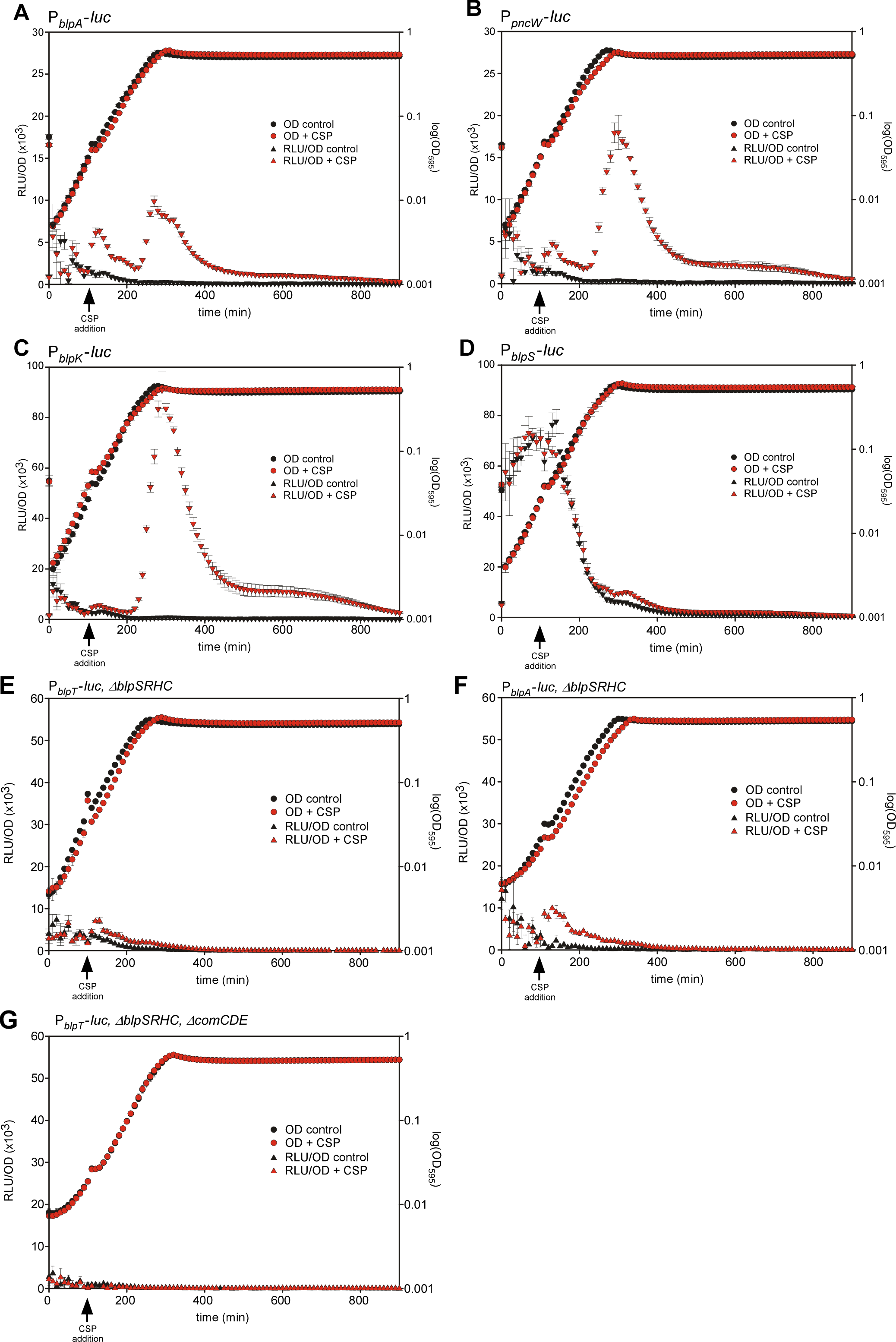

Supplement: S5 Fig — CSP induces expression from regulated blp promoters PblpA (A), PpncW (B) and PblpK (C), but does not affect expression from PblpS (D). Upon addition of CSP an immediate (weak) induction of the promoter fusions in A-C were observed, while a delayed full activation of these promoters were observed in late exponential phase. The immediate activation is independent of blpSRHC, but the delayed full activation is dependent of blpSRHC, since reporter strains for PblpT (E) or PblpA (F) with deleted blp regulatory genes (ΔblpSRHC) still show similar levels of immediate induction, but no delayed induction (compare Fig 5B with panel E and panels A with F). (G) No CSP response is observed for PblpT when blpSRHC and comCDE are deleted. For all plots, strains were grown in C+Y pH 7 with or without addition of CSP after 100 min (indicated by an arrow). Gene expression as measured by luciferase activity (RLU/OD) is shown on the left axis and growth as measured by absorbance at 595 nm (OD595) is shown on the right axis. Averages of three replicates with the standard deviation are plotted. (TIF) [file ppat.1005422.s005.tif]

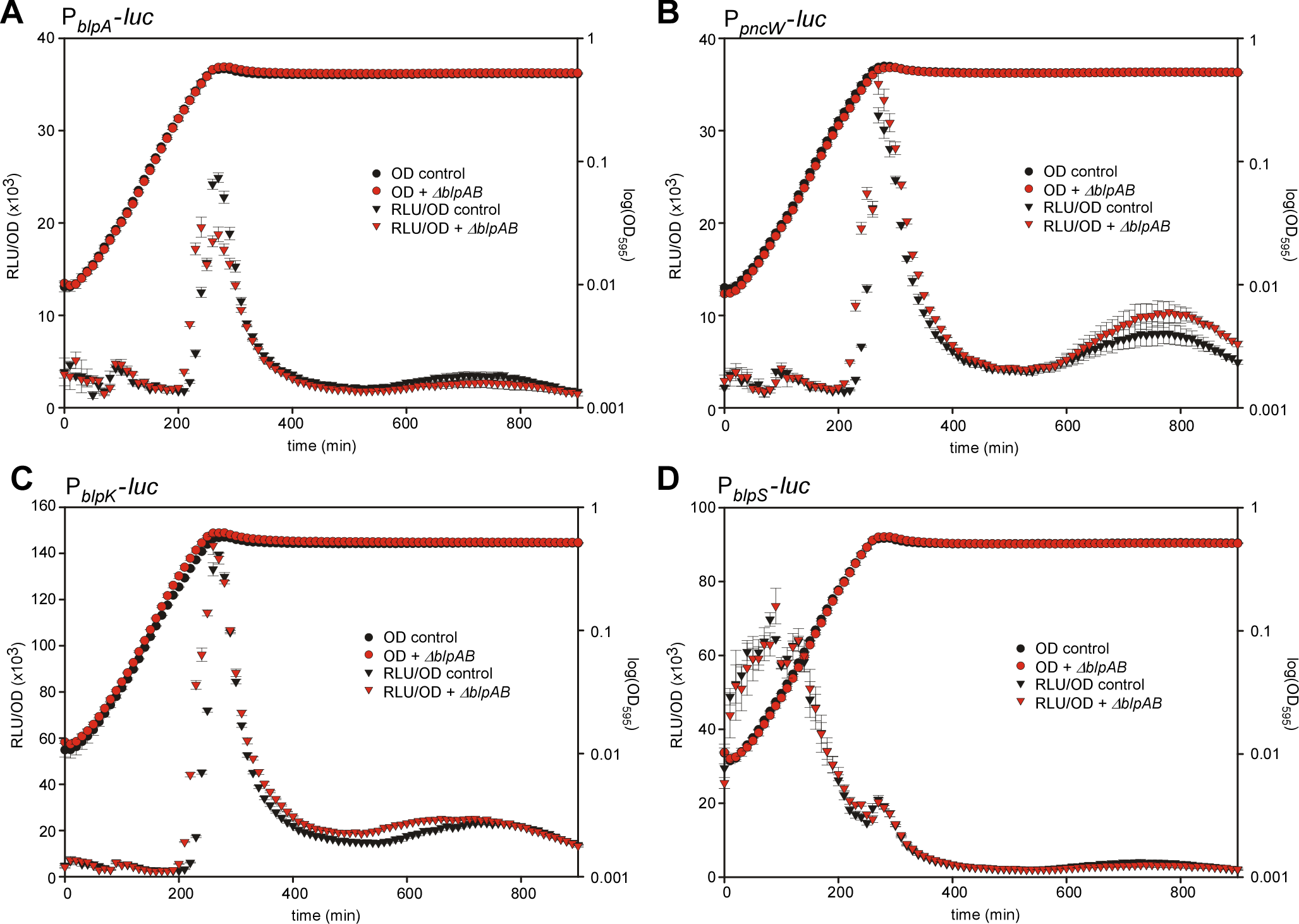

Supplement: S6 Fig — Reporter strains for the promoters PblpA (A), PpncW (B), PblpK (C) and PblpS (D) with and without deleted blpAB pseudogenes show no differences in activity when grown in C+Y pH 8. Gene expression as measured by luciferase activity (RLU/OD) is shown on the left axis and growth as measured by absorbance at 595 nm (OD595) is shown on the right axis. Averages of three replicates with the standard deviation are plotted. (TIF) [file ppat.1005422.s006.tif]

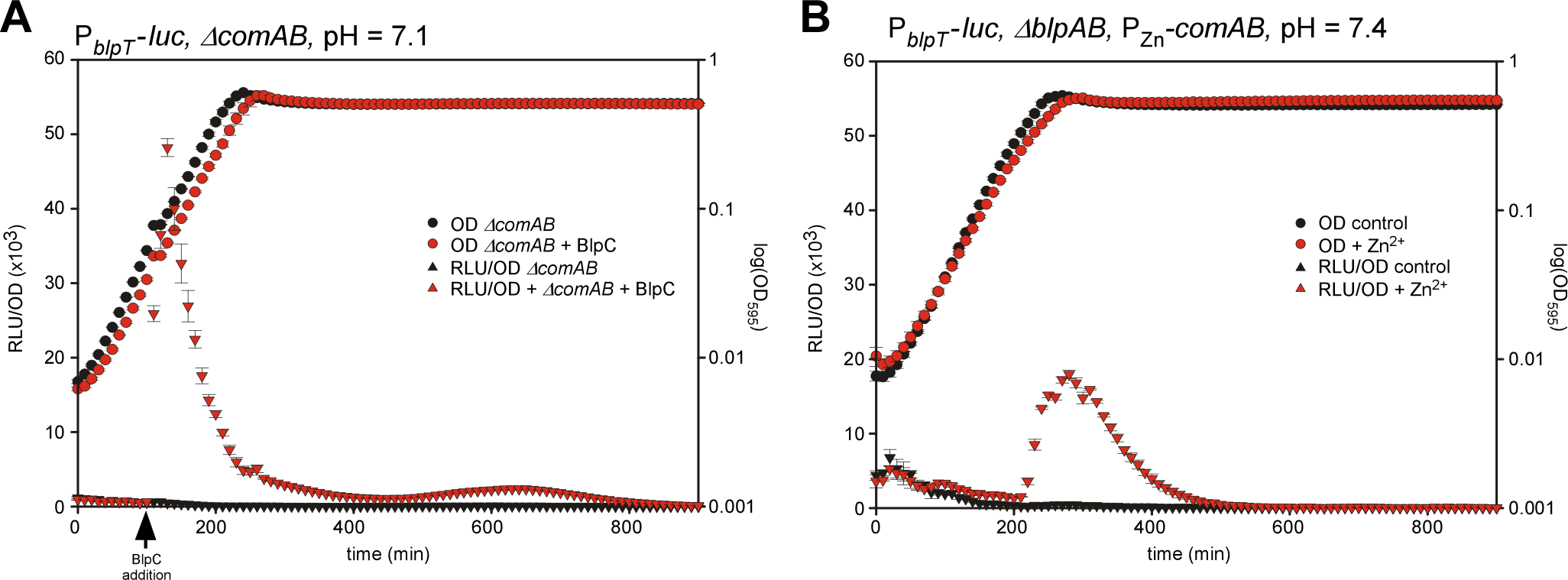

Supplement: S7 Fig — (A) blp expression can still be induced by external addition of BlpC in a comAB deletion strain. The PblpT reporter strain with deleted comAB was grown in C+Y pH 7.1. BlpC was added after 100 min, as indicated by the arrow. (B) Overexpression of comAB induces blp expression also in a blpAB deletion strain. The PblpT reporter strain with Zn2+-inducible comAB-expression was grown in C+Y pH 7.4 with or without the presence of 0.1 mM ZnCl2 and 0.01 mM MnCl2 for induction. Gene expression as measured by luciferase activity (RLU/OD) is shown on the left axis and growth as measured by absorbance at 595 nm (OD595) is shown on the right axis. Averages of three replicates with the standard deviation are plotted. (TIF) [file ppat.1005422.s007.tif]

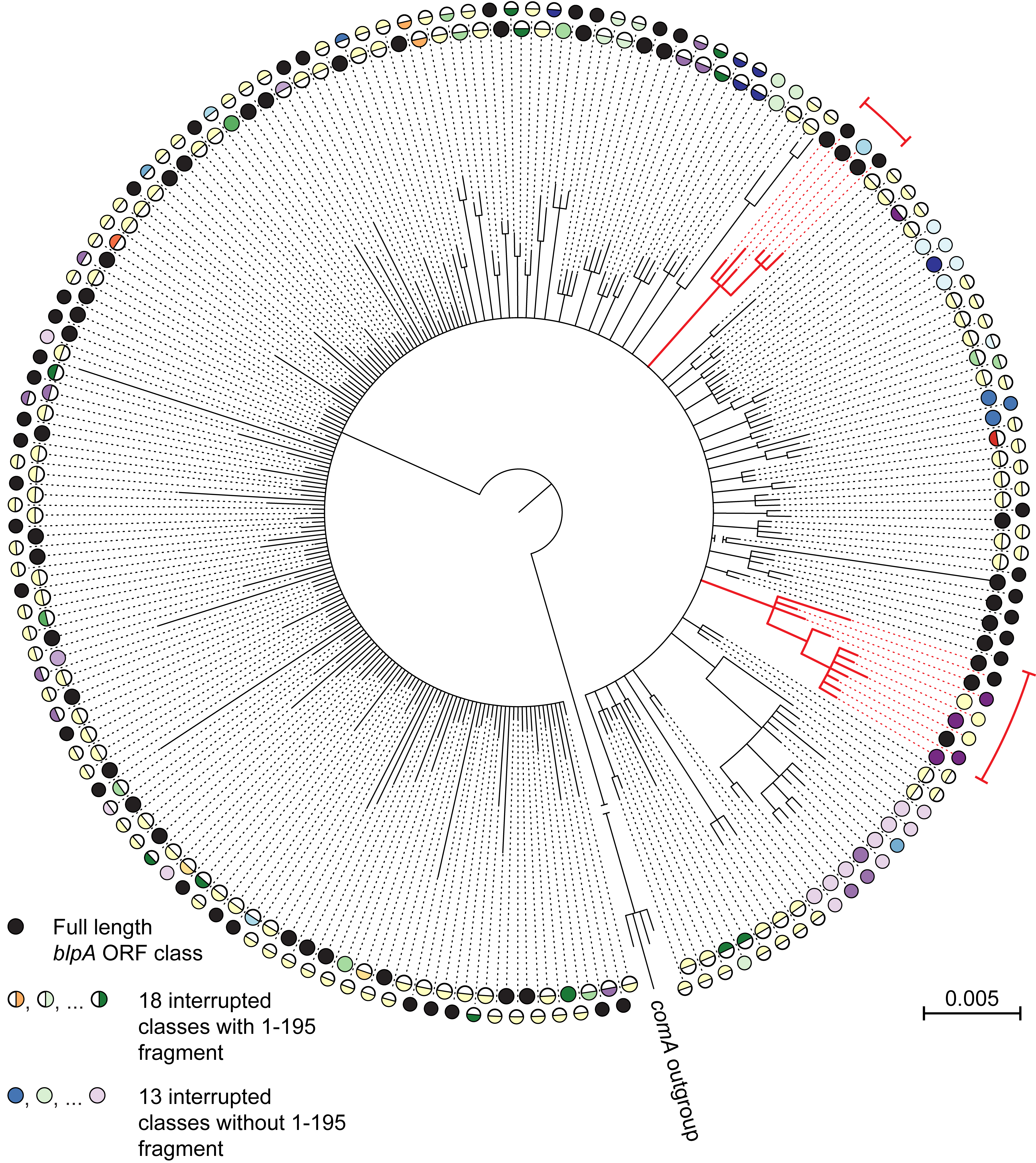

Supplement: S8 Fig — We aligned the nucleotides of the blpA locus regardless of the presence of ORFs with 4 alleles of comA as an outgroup. Sites with more than 5% gaps were removed. Using a GTR+I+G model of evolution, we used Geneious 7.1.5 and MrBayes 3.2.2 to reconstruct the phylogeny of blpA. We collapsed clades with a posterior probability of less than 0.95. In red are two clades with full-length blpA alleles as the parsimonious ancestor that contain derived interrupted blpA alleles. Alleles coding for full-length blpA are in black circles; alleles producing interrupted blpA are colored by the length classes found in Fig 1A. Alleles producing interrupted blpA with the 1–195 fragment as in Fig 1A are shown as half-colored circles. (TIF) [file ppat.1005422.s008.tif]
