# Supplementary material for: Expression of Streptococcus pneumoniae Bacteriocins Is Induced by Antibiotics via Regulatory Interplay with the Competence System
Source: PLoS Pathog. 2016 Feb 3;12(2):e1005422. doi: 10.1371/journal.ppat.1005422 (PMC4739728; doi:10.1371/journal.ppat.1005422)
Supplement: S1 Table — (DOCX) [file ppat.1005422.s009.docx]

**S1 Table**. Transcriptional response of *blp* genes of *S. pneumoniae* D39 after exposure to sub-lethal level of antibiotics as determined by RNA sequencing^*^

| Gene | Name^†^ | HPUra | Kanamycin | Ciprofloxacin | Hydroxyurea | Rifampicin |
| --- | --- | --- | --- | --- | --- | --- |
| Blp genes |  |  |  |  |  |  |
| SPD_0046 | *blpK* | - | - | 7,9 | - | - |
| SPD_0047 | - | - | - | 4,4 | - | - |
| SPD_0466 | *blpT* | - | - | - | - | - |
| SPD_0467 | *blpS* | - | - | - | - | - |
| SPD_0468 | *blpR* | - | - | - | - | - |
| SPD_0469 | *blpH* | - | - | - | - | - |
| SPD_0470 | *blpC* | - | - | - | - | - |
|  | *'blpB* | - | - | - | - | - |
|  | *blpB'* | - | - | - | - | - |
|  | *'blpA* | - | - | - | - | - |
|  | *blpA'* | 2,9 | - | - | - | - |
|  | *pncW* | 4,8 | 4,7 | 6,8 | 14,1 |  |
| SPD_0473 | *blpY* | 5,6 | - | 6,0 | 11,3 | - |
| SPD_0474 | *blpZ* | 7,3 | - | 8,2 | 12,6 | - |
| SPD_0475 | *pncP* | 5,8 | - | 4,9 | 7,8 | - |
|  |  |  |  |  |  |  |
| Com genes (early)^‡^ | |  |  |  |  |  |
| SPD_0049 | *comA* | 65,0 | 57,8 | 332,3 | 69,8 | - |
| SPD_0050 | *comB* | 58,7 | 51,0 | 461,5 | 70,7 | - |
| SPD_2065 | *comC1* | 18,4 | 24,1 | 20,2 | 5,3 | - |
| SPD_2064 | *comD* | 58,7 | 50,2 | 55,7 | 12,4 | - |
| SPD_2063 | *comE* | 55,6 | 48,1 | 43,5 | 9,4 | - |
| SPD_0014 | *comX* | 86,8 | 107,7 | 366,5 | 28,8 | - |
|  |  |  |  |  |  |  |
| Com genes (late) ^‡^ | |  |  |  |  |  |
| SPD_1711 | *ssbB* | 1120,3 | 61,8 | 690,6 | 161,9 | - |
| SPD_2028 | *cbpD* | 160,0 | 10,0 | 370,0 | 32,3 | - |
| SPD_0975 | *radC* | 93,8 | 10,9 | 50,2 | 7,9 | - |
| SPD_1863 | *cglA* | 61,8 | 5,5 | 991,0 | 45,7 | - |
| SPD_1862 | *cglB* | 275,7 | 14,0 | 666,7 | 73,7 | - |
| SPD_1861 | *cglC* | 196,7 | 9,3 | 463,5 | 33,3 | - |
| SPD_1860 | *clgD* | 301,6 | 13,0 | 394,4 | 43,4 | - |

^*^The fold-changes between the antibiotic-exposed samples and the control sample (normal growth, no exposure to antibiotics) are given in all cases where the q-values < 0.05. A dash (-) denotes no significant regulation. For this experiment, cells were harvested in the early growth phase, when they had reached 1/3 of the maximal OD_600_ [1].

^†^ Names of putative bacteriocin and immunity genes are given according to Boogardt et al. [2].

^‡^Selected early- and late-competence genes shown [3,4].

**References**

1. Slager J, Kjos M, Attaiech L, Veening J-W. Antibiotic-induced replication stress triggers bacterial competence by increasing gene dosage near the origin. Cell. 2014;157: 395–406. doi:10.1016/j.cell.2014.01.068

2. Bogaardt C, Tonder AJ van, Brueggemann AB. Genomic analyses of pneumococci reveal a wide diversity of bacteriocins – including pneumocyclicin, a novel circular bacteriocin. BMC Genomics. 2015;16: 554. doi:10.1186/s12864-015-1729-4

3. Dagkessamanskaia A, Moscoso M, Hénard V, Guiral S, Overweg K, Reuter M, et al. Interconnection of competence, stress and CiaR regulons in *Streptococcus pneumoniae*: competence triggers stationary phase autolysis of *ciaR* mutant cells. Mol Microbiol. 2004;51: 1071–1086. doi:10.1111/j.1365-2958.2003.03892.x

4. Peterson SN, Sung CK, Cline R, Desai BV, Snesrud EC, Luo P, et al. Identification of competence pheromone responsive genes in *Streptococcus pneumoniae* by use of DNA microarrays. Mol Microbiol. 2004;51: 1051–1070. doi:10.1046/j.1365-2958.2003.03907.x
