# Supplementary material for: Expression of Streptococcus pneumoniae Bacteriocins Is Induced by Antibiotics via Regulatory Interplay with the Competence System
Source: PLoS Pathog. 2016 Feb 3;12(2):e1005422. doi: 10.1371/journal.ppat.1005422 (PMC4739728; doi:10.1371/journal.ppat.1005422)
Supplement: S2 Table — (DOCX) [file ppat.1005422.s010.docx]

**S2 Table**. Strains and plasmids used in this study.

|  | Characteristics* | References |
| --- | --- | --- |
| *S. pneumoniae* strains |  |  |
| D39 | Serotype 2 strain | [1] |
| DLA3 | D39, *ΔbgaA*::(P*_ssbB_-luc*, *tet*^R^) | [2] |
| D-PEP23 | D39, *Δcep::(P3-luc, spc^R^*) | [3] |
| FB121 | D39, *ΔbgaA::tet*^R^, *Δcep::*(*P_blpA_-LGZ, spc^R^*) | This study |
| FB123 | D39, *ΔbgaA::tet*^R^, *Δcep::*(*P_blpU_-LGZ, spc^R^*) | This study |
| FB125 | D39, *ΔbgaA::tet*^R^, *Δcep::*(*P_blpT_-LGZ, spc^R^*) | This study |
| FB127 | D39, *ΔbgaA::tet*^R^, *Δcep::*(*P_blpS_-LGZ, spc^R^*) | This study |
| FB129 | D39, *ΔbgaA::tet*^R^, *Δcep::*(*P_blpV1_-LGZ, spc^R^*) | This study |
| FB131 | D39, *ΔbgaA::tet*^R^, *Δcep::*(*P_blpA_-LGZ, spc^R^*), *ΔblpSRHC::ery^R^* | This study |
| FB133 | D39, *ΔbgaA::tet*^R^, *Δcep::*(*P_blpU_-LGZ, spc^R^*), *ΔblpSRHC::ery^R^* | This study |
| FB135 | D39, *ΔbgaA::tet*^R^, *Δcep::*(*P_blpT_-LGZ, spc^R^*), *ΔblpSRHC::ery^R^* | This study |
| FB137 | D39, *ΔbgaA::tet*^R^, *Δcep::*(*P_blpS_-LGZ, spc^R^*), *ΔblpSRHC::ery^R^* | This study |
| FB139 | D39, *ΔbgaA::tet*^R^, *Δcep::*(*P_blpV1_-LGZ, spc^R^*), *ΔblpSRHC::ery^R^* | This study |
| FB141 | D39, *Δcep::*(*P_blpT_-LGZ, spc^R^*), *ΔblpSRHC::ery^R^* | This study |
| FB151 | D39, *ΔbgaA::tet*^R^, *Δcep::*(*P_blpT_-LGZ, spc^R^*), *ΔcomCDE::cam^R^* | This study |
| FB153 | D39, *ΔbgaA::tet*^R^, *Δcep::*(*P_blpT_-LGZ, spc^R^*), *ΔcomA::ery^R^* | This study |
| FB155 | D39, *Δcep::*(*P_blpT_-LGZ, spc^R^*), *ΔblpSRHC::ery^R^*, *ΔbgaA*::(P*_blpS_*-*blpSRH, tet^R^)* | This study |
| FB194 | D39, *Δcep::*(*P_blpT_-LGZ, spc^R^*), *ΔblpSRHC::ery^R^*, *ΔcomCDE::cam^R^* | This study |
| FB197 | D39, *ΔbgaA::tet*^R^, *Δcep::*(*P_blpT_-LGZ, spc^R^*), *ΔcomB::ery^R^* | This study |
| FB200 | D39, *ΔbgaA::tet*^R^, *Δcep::*(*P_blpT_-LGZ, spc^R^*), *ΔcomAB::ery^R^* | This study |
| FB204 | D39, *Δcep::*(*P_blpT_-LGZ, spc^R^*), *ΔbgaA*::(P_Zn_-*comA, tet^R^)* | This study |
| FB207 | D39, *Δcep::*(*P_blpT_-LGZ, spc^R^*), *ΔbgaA*::(P_Zn_-*comB, tet^R^)* | This study |
| FB210 | D39, *Δcep::*(*P_blpT_-LGZ, spc^R^*), *ΔbgaA*::(P_Zn_-*comAB, tet^R^)* | This study |
| FB213 | D39, *Δcep::*(*P_blpT_-LGZ, spc^R^*), *ΔbgaA*::(P_Zn_-*comAB, tet^R^), ΔcomAB::ery^R^* | This study |
| FB216 | D39, *Δcep::*(*P_blpT_-LGZ, spc^R^*), *ΔbgaA*::(P_Zn_-*comAB, tet^R^), ΔcomCDE::cam^R^* | This study |
| FB219 | D39, *Δcep::*(*P_blpT_-LGZ, spc^R^*) | This study |
| FB231 | D39, *Δcep::*(*P_blpT_-LGZ, spc^R^*), *ΔbgaA*::(P*_ssbB_-rfp, tet^R^*) | This study |
| MK110 | D39, *comCDE*, *ery*^R^ | [2] |
| MK303 | D39, *ΔbgaA::tet^R^* | This study |
| MK304 | D39, *ΔbgaA::tet*^R^, *ΔblpSRHC::ery^R^* | This study |
| MK436 | D39, *ΔbgaA::tet*^R^, *Δcep::*(*P_blpK_-LGZ, spc^R^*), *ΔblpA’B’*::*ery* | This study |
| MK437 | D39, *ΔbgaA::tet*^R^, *Δcep::*(*P_blpT_-LGZ, spc^R^*), *ΔblpA’B’*::*ery* | This study |
| MK438 | D39, *ΔbgaA::tet*^R^, *Δcep::*(*P_blpS_-LGZ, spc^R^*), *ΔblpA’B’*::*ery* | This study |
| MK439 | D39, *ΔbgaA::tet*^R^, *Δcep::*(*P_pncW_-LGZ, spc^R^*), *ΔblpA’B’*::*ery* | This study |
| MK440 | D39, *ΔbgaA::*(P_Zn_-*comAB*, tet^R^), *Δcep::*(*P_blpT_-LGZ, spc^R^*), *ΔblpA’B’*::*ery* | This study |
| MK441 | D39, *ΔbgaA::tet*^R^, *Δcep::*(*P_blpA_-LGZ, spc^R^*), *ΔblpA’B’*::*ery* | This study |
|  |  |  |
| Plasmids |  |  |
| pJWV25 | *amp^R^, bgaA’, tetR, P_Zn_-gfp, ’bgaA* | [4] |
| pJWV100 | *amp^R^, bgaA’, tetR,* (no promoter) *gfp, ’bgaA* | [5] |
| pPEP1 | *cam^R^*_,_ *cep’*, *spc^R^*-MCS,*‘cep* | [3] |
| pPEP1-LGZ | *cam^R^*, *cep’*, *spc^R^*, (no promoter)-*luc*-*gfp*-*lacZ*,*‘cep* | This study |
| pPEP1-P*_blpT_*-LGZ | *cam^R^*, *cep’*, *spc^R^*-P*_blpT_*-*luc*-*gfp*-*lacZ*,*‘cep* | This study |
| pPEP1-P*_blpU_*-LGZ | *cam^R^*, *cep’*, *spc^R^*-P*_blpU_*-*luc*-*gfp*-*lacZ*,*‘cep* | This study |
| pPEP1-P*_blpS_*-LGZ | *cam^R^*, *cep’*, *spc^R^*-P*_blpS_*-*luc*-*gfp*-*lacZ*,*‘cep* | This study |
| pPEP1-P*_blpA_*-LGZ | *cam^R^*, *cep’*, *spc^R^*-P*_blpA_*-*luc*-*gfp*-*lacZ*,*‘cep* | This study |
| pPEP1-P*_blpV1_*-LGZ | *cam^R^*, *cep’*, *spc^R^*-P*_blpV1_*-*luc*-*gfp*-*lacZ*,*‘cep* | This study |
| pMK11 | *amp^R^*, *bgaA’*, *tet^R^*, P_Zn_, *’bgaA* | This study |
| pMK11-P*_Zn_*-*comA* | *amp^R^*, *bgaA’*, *tet^R^*, P_Zn_-*comA*, *’bgaA* | This study |
| pMK11-P*_Zn_*-*comB* | *amp^R^*, *bgaA’*, *tet^R^*, P_Zn_-*comB*, *’bgaA* | This study |
| pMK11-P*_Zn_*-*comAB* | *amp^R^*, *bgaA’*, *tet^R^*, P_Zn_-*comAB*, *’bgaA* | This study |
| pLA21 | *amp^R^*, *bgaA’*, *tet^R^*, P*_ssbB_*-rfp, *’bgaA* | [6] |

* In the constructs description, “LGZ” is short for the tripartite reporter cassette containing firefly luciferase, *luc* (L), *gfp* (G) and β-galactosidase , *lacZ* (Z).

**References**

1. Avery OT, Macleod CM, McCarty M. Studies on the chemical nature of the substance inducing transformation of pneumococcal types: induction of transformation by a deoxyribonucleic acid fraction isolated from pneumococcus type III. J Exp Med. 1944;79: 137–158.

2. Slager J, Kjos M, Attaiech L, Veening J-W. Antibiotic-induced replication stress triggers bacterial competence by increasing gene dosage near the origin. Cell. 2014;157: 395–406. doi:10.1016/j.cell.2014.01.068

3. Sorg RA, Kuipers OP, Veening J-W. Gene expression platform for synthetic biology in the human pathogen *Streptococcus pneumoniae*. ACS Synth Biol. 2014;4: 228–239. doi:10.1021/sb500229s

4. Eberhardt A, Wu LJ, Errington J, Vollmer W, Veening J-W. Cellular localization of choline-utilization proteins in *Streptococcus pneumoniae* using novel fluorescent reporter systems. Mol Microbiol. 2009;74: 395–408. doi:10.1111/j.1365-2958.2009.06872.x

5. Overkamp W, Beilharz K, Detert Oude Weme R, Solopova A, Karsens H, Kovács ÁT, et al. Benchmarking various green fluorescent protein variants in *Bacillus subtilis*, *Streptococcus pneumoniae*, and *Lactococcus lactis* for live cell imaging. Appl Environ Microbiol. 2013;79: 6481–6490. doi:10.1128/AEM.02033-13

6. Attaiech L, Minnen A, Kjos M, Gruber S, Veening J-W. The ParB-*parS* chromosome segregation system modulates competence development in *Streptococcus pneumoniae*. MBio. 2015;6: e00662–15. doi:10.1128/mBio.00662-15.
