# Supplementary material for: Expression of Streptococcus pneumoniae Bacteriocins Is Induced by Antibiotics via Regulatory Interplay with the Competence System
Source: PLoS Pathog. 2016 Feb 3;12(2):e1005422. doi: 10.1371/journal.ppat.1005422 (PMC4739728; doi:10.1371/journal.ppat.1005422)
Supplement: S3 Table — (DOCX) [file ppat.1005422.s011.docx]

**S3 Table**. Oligonucleotides used in this study

| Name | Sequence (5’ 🡪 3’)* |
| --- | --- |
| Blp_SRHC_dn_F_NotI | GCGGCCGCAAGATAATAAATAGTTATAGAGTGTTATC |
| Blp_SRHC_dn_R | GGATGAATTTGGCTACCATAATACC |
| Blp_SRHC_up_F | TCTTTAACTACCTCTTGCTCTTCCAG |
| Blp_SRHC_up_R_BamHI | GGATCCACCCAATCCTAAACAGTCTTTGAAAAC |
| BlpA_F_NheI_NotI | GAACGCTAGCGCGGCCGCTGAATTGTCATCGAAACG |
| BlpA_R_BglII | AGATCTTCTCGCATCTATTTGAGGAACAAATG |
| blpA-up-F-BamHI | ACGTGGATCCCTATTTGAGGAACAAATGTACGT |
| blpA-up-R | CTTAAAGAAGGCGTGTTGAAG |
| blpB-down-F | TTCGCCATCTCATCCTAGTG |
| blpB-down-R-NotI | ATGCGCGGCCGCAAGATAAAATTTTAACCCATTCTG |
| comA1 | GCGCAATGAAGCTCATCTCC |
| comA2+AscI | TAATGGCGCGCCAAGCTACACCGCAGTCCATC |
| comA3+NotI | AATAGCGGCCGCTGGATCAGGGCAAGATTGTC |
| comA4 | TACCTGACTCTGCCTCCAAG |
| comB_down_F+NotI | AGCGCGGCCGCTTAGATCAATTTTTGAACAAAGAG |
| comB_down_R | CTGTTAGTTCACCTAGTTCTC |
| comB_up_R+AscI | ACGTGGCGCGCCGACGATTATAAAACTCCGCAC |
| end-comA+SpeI | ATGCACTAGTCTTTCTAGCTATTGACCAAATGG |
| end-comB+SpeI | ATGCACTAGTCTCTAAAAACACGAACATTACTC |
| eryR-down+NotI | GTCAGCGGCCGCGTAGGCGCTAGGGACCTC |
| Ery-For-BamHI | ACGTCGGATCCAAGAAGGAGTGATTACATGAAC |
| eryR-up_F_BamHI | CGATGGATCCAACAGCAAAGAATGGCGGAAACGT |
| lacZ_F_EcoRI_BglII | GCGAATTCATGAGATCTTAGTTAAGGAGGCAAATATG |
| Luc_F_BglII_AseI_EcoRI | GCGAATTCGATTAATCAGATCTAATTAGCTGAAGGAGGAATAATG |
| PblpS_F_NheI_NotI | GAACGCTAGCGCGGCCGCTGTTAGTTTATCACTTTTAATTC |
| PblpS_R_BglII | AGATCTTGTTTTCTGTGTCTGAATAATCATATG |
| PblpT_F_NotI_NheI | GAACGCTAGCGCGGCCGCCCTAAACAGTCTTTGAAAAC |
| PblpT_R_BglII | AGATCTAATCAAAGTATGAGCTCTTTTGATG |
| PblpU_F_NotI_NheI | GAACGCTAGCGCGGCCGCTCATTAAAACTTCCTG |
| PblpU-R+BamHI | CGATGGATCCCATTATCCATAACAGAAAATTGTG |
| pr27 | ACGGTACCGCATGCTGCTGATACGGCGGTCAATG |
| pr28 | GCACTAGTGCTCAGCTATGAATTCTCATATTTGCCTCCTTAAGAT |
| Pspd_0046-F+NheI+NotI | GAACGCTAGCGCGGCCGCGTATTATCAATCATGAACTAGGA |
| Pspd_0046-R+BglII | ACGTCAGATCTAAGCAAGCATCTCAGTATCCA |
| sPG20_eryR+NotI | AGCTGCGGCCGCTTATTTCCTCCCGTTAAATA |
| start-comA+EcoRI | ATGCGAATTCTTAGGGAAAAGGAGATGAATATG |
| start-comB+EcoRI | ATGCGAATTCAGAGGAGAGGATGAAACCAGA |
| trmp-F+AscI | GCATGGCGCGCCGGATTTTTGTGAGCTTGGA |

* Restriction sites are underlined.
